# ATP Maintenance via Two Types of ATP Regulators Mitigates Pathological Phenotypes in Mouse Models of Parkinson's Disease

**DOI:** 10.1016/j.ebiom.2017.07.024

**Published:** 2017-07-25

**Authors:** Masaki Nakano, Hiromi Imamura, Norio Sasaoka, Masamichi Yamamoto, Norihito Uemura, Toshiyuki Shudo, Tomohiro Fuchigami, Ryosuke Takahashi, Akira Kakizuka

**Affiliations:** a Laboratory of Functional Biology, Kyoto University Graduate School of Biostudies, Kyoto 606-8501, Japan; b Department of Nephrology, Kyoto University Graduate School of Medicine, Kyoto 606-8507, Japan; c Department of Neurology, Kyoto University Graduate School of Medicine, Kyoto 606-8507, Japan; d Daito Chemix, Ishibashi-cho, Fukui-city, Fukui 910-3137, Japan

**Keywords:** ATP, Parkinson's disease, ER stress, Dopaminergic neurons, Mitochondria, α-Synuclein

## Abstract

Parkinson's disease is assumed to be caused by mitochondrial dysfunction in the affected dopaminergic neurons in the brain. We have recently created small chemicals, KUSs (Kyoto University Substances), which can reduce cellular ATP consumption. By contrast, agonistic ligands of ERRs (estrogen receptor-related receptors) are expected to raise cellular ATP levels via enhancing ATP production. Here, we show that esculetin functions as an ERR agonist, and its addition to culture media enhances glycolysis and mitochondrial respiration, leading to elevated cellular ATP levels. Subsequently, we show the neuroprotective efficacies of KUSs, esculetin, and GSK4716 (an ERRγ agonist) against cell death in Parkinson's disease models. In the surviving neurons, ATP levels and expression levels of α-synuclein and CHOP (an ER stress-mediated cell death executor) were all rectified. We propose that maintenance of ATP levels, by inhibiting ATP consumption or enhancing ATP production, or both, would be a promising therapeutic strategy for Parkinson's disease.

## Highlights

• We describe two types of ATP regulators: one limits ATP consumption, while the other enhances ATP production.
• ATP regulators maintain ATP levels and protect neuronal cells from ER stress and eventual cell death.
• ATP regulators mitigate disease phenotypes in mouse models of Parkinson's disease.

ATP is essential in human activities and health. We hypothesize that maintaining ATP levels may help to preserve vulnerable brain cells in neurodegenerative diseases like Parkinson's disease (PD). For this purpose, we developed and tested two types of chemical compounds, one for limiting ATP consumption and the other for enhancing ATP production. These chemicals, which we call “ATP regulators”, indeed mitigated the pathological phenotypes in two mouse models of PD, indicating that ATP regulation is a promising therapeutic strategy for currently incurable diseases, such as PD, and possibly other neurodegenerative diseases.

## 1 Introduction

The human brain constitutes only 2–3% of the total body weight but monopolizes about 15% of the total blood flow, and it consumes about 20% of the total oxygen inhaled by the lungs. Simply put, the brain needs a lot of energy, namely ATP, for its functions and survival. Indeed, a decrease in ATP production in certain area(s) of the brain or the central nervous system can lead to neuronal cell death in the affected area(s) and thus cause ischemic or degenerative neuronal diseases.

Parkinson's disease is the second-most frequent neurodegenerative disease, with an incidence of approximately one per 1000 individuals. Parkinson's disease is caused by gradual cell death of dopaminergic neurons in the substantia nigra, and therapeutic chemicals or drugs that prevent this cell death are currently not available. In the early 1980s, certain numbers of drug users near San Francisco were observed manifesting Parkinson's disease phenotypes (Langston et al., 1983). The examination clarified that they suffered from accidental intoxication by 1-methyl-4-phenyl-1,2,3,6-tetrahydropyridine (MPTP), which was a contaminant in the drug they used. Later, it was found that 1-methyl-4-phenylpyridinium (MPP +), a metabolite of MPTP, but not MPTP itself, is specifically incorporated into dopaminergic neurons in the substantia nigra, where it inhibits mitochondrial complex I, suppresses ATP production, and ultimately kills the dopaminergic neurons in vivo, not only in humans but also in other animals, e.g. mice and rats (Dauer and Przedborski, 2003, Davis et al., 1979, Langston et al., 1983, Ramsay et al., 1991). These results have strongly implicated mitochondrial dysfunction or ATP decrease as a pathological mechanism in Parkinson's disease. Since then, MPTP has been widely used to experimentally create animal models of Parkinson's disease.

The most well-known, if not invariable, pathological hallmark of Parkinson's disease is the presence of Lewy bodies, protein aggregates composed of α-synuclein, in dopaminergic neurons in the affected brain region (Dauer and Przedborski, 2003, Halliday et al., 2014, Polymeropoulos et al., 1997, Spillantini et al., 1997). Similar α-synuclein aggregates are also observed in cortical neurons in dementia with Lewy bodies and in glial cells in multisystem atrophy (Dauer and Przedborski, 2003, Halliday et al., 2014). These neurodegenerative diseases are collectively referred to as “α-synucleinopathies” (McCann et al., 2014). From a genetic point of view, 18 genetic loci have been linked to familial Parkinson's disease, and are named PARK1 to PARK18 (Klein and Westenberger, 2012, Lin and Farrer, 2014). PARK1 encodes α-synuclein itself (Klein and Westenberger, 2012, Lin and Farrer, 2014, Polymeropoulos et al., 1997). PARK2, PARK6, and PARK17 encode Parkin, PINK1, and VPS35, respectively (Kitada et al., 1998, Klein and Westenberger, 2012, Lin and Farrer, 2014, Sharma et al., 2012, Valente et al., 2004, Vilariño-Güell et al., 2011, Zimprich et al., 2011). It is noteworthy that both Parkin and PINK1 collaboratively function to maintain mitochondrial function (Pickrell and Youle, 2015), and that VPS35 also operates to maintain mitochondrial function (Tang et al., 2015, Wang et al., 2016). Furthermore, it has been shown that genetic manipulation to maintain mitochondrial functions renders mice resistant to MPTP-induced Parkinson's disease (Hasegawa et al., 2016a, Mudò et al., 2012). These lines of evidence again indicate that dysfunctional mitochondria and ATP decrease are underlying factors in the etiology of Parkinson's disease, and suggest a potential link between the production of α-synuclein aggregates and ATP decrease.

In addition to Parkinson's disease, abnormal protein aggregates are also observed in several other neurodegenerative disorders, e.g. Alzheimer's disease, amyotrophic lateral sclerosis (ALS), polyglutamine diseases, etc., suggesting that common mechanisms underlie these disorders (Kakizuka, 1998). During long-term analyses of the molecular bases of neuronal cell death in neurodegenerative disorders (Ikeda et al., 1996, Kakizuka, 1998, Kawaguchi et al., 1994), we found that ATP consumption by valosin-containing protein (VCP) was profoundly involved in neurodegenerative phenotypes (Higashiyama et al., 2002, Manno et al., 2010). It is notable that *VCP* mutations have been implicated in IBMPFD (inclusion body myopathy with Paget disease of bone and frontotemporal dementia) (Watts et al., 2004) and rare cases of familial ALS (Johnson et al., 2010). In our analyses, all examined mutated VCP proteins had elevated ATPase activities, and the relative increase in activity levels appeared to be correlated with the severity of the clinical phenotypes (Manno et al., 2010). From these results, we hypothesized that specific inhibitors of VCP ATPase activity might ameliorate the disease phenotypes of familial VCP diseases as well as neuronal cell death in other diseases.

Previously, we showed that PGC1β, a member of the PGC1 family, functions as a protein ligand specific for estrogen receptor-related receptors (ERRs) (Kamei et al., 2003). ERRs belong to the nuclear receptor superfamily and mediate transcription for mitochondrial biogenesis or enhancement of energy expenditure (Alaynick et al., 2007, Audet-walsh and Giguère, 2015, Dufour et al., 2007, Huss et al., 2004). Indeed, transgenic expression of PGC1β produced phenotypes of high-energy expenditure: PGC1β mice are big eaters but thin (Kamei et al., 2003). These results indicate that natural or synthetic ERR ligands could enhance ATP production.

Recently, we successfully created small chemicals that are able to suppress the ATPase activity of VCP (Ikeda et al., 2014). Under various stress conditions in cultured cells, the chemicals (KUSs) were able to significantly maintain cellular ATP levels, and consequently suppress ER stress and cell death (Ikeda et al., 2014, Nakano et al., 2016). In addition, KUSs showed significant efficacies in preventing retinal neuronal cell death in in vivo mouse models of retinitis pigmentosa, glaucoma, and ischemic retinal disease (Hasegawa et al., 2016b, Hata et al., 2017, Ikeda et al., 2014, Nakano et al., 2016). In this manuscript, we show that two classes of small chemicals, one for limiting ATP consumption, and the other for enhancing ATP production via ERRs, possess strong efficacies in maintaining ATP levels and in protecting neuronal cells from death in both in vitro cell culture and in vivo mouse models of Parkinson's disease.

## 2 Materials and Methods

### 2.1 Experimental Design

Our objectives were to evaluate the efficacy of ATP maintenance via two types of ATP regulators in MPTP-induced and rotenone-induced Parkinson's disease models. We investigated two classes of small chemicals, one for preventing ATP consumption by VCP (KUSs), and the other for enhancing ATP production via ERRs (esculetin). Their efficacies were first determined by assessing the inhibition of neuronal cell death, ATP levels, and ER stress (CHOP expression) in in vitro cell culture models of Parkinson's disease. We also examined their efficacies in in vivo mouse models of Parkinson's disease by monitoring behavior, ATP levels, and histology. Sample numbers and sizes, the composition of replicates, and doses of compounds were based on pilot experiments and prior knowledge of cultured cells or mouse experiments (Hasegawa et al., 2016b, Ikeda et al., 2014, Nakano et al., 2016). Randomization of animals in the study was based on initial assessments of locomotor activity with a rotarod test and body weight to ensure an equal distribution in each group. Group sizes were set to enable statistical analyses in detection of locomotor activity (more than five per group), histological analyses (more than three per group), or measurement of ATP levels in mouse brain (fourteen or fifteen per group). ATP levels were evaluated using two methods (classic luciferase activity-based methods and imaging of ATeam (Imamura et al., 2009, Nakano et al., 2011, Tsuyama et al., 2013). All rules were predefined in advance. All data were included. Cell culture experiments were performed more than three times. The final end point before sacrifice in mouse experiments was when Parkinson's disease symptoms fully appeared. All animal studies containing the final end points before sacrifice were approved by the Animal Care and Use Committee of Kyoto University.

### 2.2 Cell Culture

PC12 cells were cultured in low glucose Dulbecco's modified Eagle's medium (DMEM) (Nacalai Tesque, Kyoto, Japan), supplemented with 10% fetal bovine serum (FBS) (Sigma, St. Louis, MO, USA) and 5% horse serum (HS) (Sigma), and maintained at 37 °C in a humidified atmosphere of 10% CO_2_. HEK293A cells were cultured in high glucose DMEM (Nacalai Tesque) supplemented with 10% FBS (Sigma), and maintained at 37 °C in a humidified atmosphere of 5% CO_2_.

### 2.3 ERR Reporter Assay

HEK293A cells were cultured at 37 °C in high glucose DMEM (Nacalai Tesque), supplemented with 10% FBS (Sigma). Cells were transfected with plasmid DNA using Polyethylenimine “Max” (Polysciences Inc., Warrington, PA, USA). HEK293A cells were cultured in a 24-well plate, and co-transfected with 0.2 μg each of pTk-(GalRE) × 4-Luc and pCMX-β-galactosidase, along with 0.2 μg of one of the following: pCMX-Gal4 (as a control); pCMX-Gal4-hERRα; pCMX-Gal4-hERRβ; pCMX-Gal4-hERRγ. 24 h after transfection, KUS121, KUS187, esculetin, GSK4716, or GSK5182 were added to the culture medium, and cells were incubated for an additional 24 h. Then, whole cell lysates were prepared, and luciferase and β-galactosidase assays were carried out, as described previously (Saitou et al., 1994, Sasaoka et al., 2014). The observed luciferase activities were normalized by the β-galactosidase activities to compensate for different transfection efficiencies (Saitou et al., 1994, Sasaoka et al., 2014).

### 2.4 Assay of Neurotoxicity Induced by Mitochondrial Complex Inhibitors

1 × 10^5^ PC12 cells were cultured in 6-well plates at 37 °C in DMEM supplemented with 10% FBS (Sigma) and 5% HS (Sigma). To stimulate differentiation, the culture medium was replaced with DMEM supplemented with 1% FBS, 1% HS, and 50 ng/ml NGF (Alomone Labs, Jerusalem, Israel), and cells were cultured for 24 h. During differentiation, KUSs and esculetin were included in test wells. Then mitochondrial respiratory chain complex inhibitors such as MPP + (75 μM) (Sigma), rotenone (10 nM) (Sigma), metformin (3 mM) (Sigma), antimycin (100 nM) (Sigma), and oligomycin (0.01 μg/ml) (Sigma) were added. After an additional incubation for 24 h, ATP assays and western blots were performed. In parallel assays, after 28 h, cell viability assays using trypan blue (Dettmer et al., 2015) and an LDH cytotoxicity assay were performed (Sharpe et al., 2015).

### 2.5 Glucose Consumption

In order to determine the levels of glucose, cell culture supernatants were collected and assayed for glucose levels with a glucose assay kit (Wako, Tokyo, Japan), following the manufacturer's protocol. Glucose concentrations in medium samples taken at the start and end of the experiments were used to determine cellular uptake. Glucose consumption was calculated, and normalized by the cell numbers.

### 2.6 ATP Assay

ATP levels in cell lysates or brain extracts were measured with luciferase chemiluminescence-based ATP assay reagent for cells (Toyo B-net, Tokyo, Japan), following the manufacturer's protocol. Briefly, cell lysates were mixed with Glo-Lysis buffer (Promega Corporation, Madison, WI, USA) by shaking slightly and letting the mixture sit for 5 min. Brain sample was homogenized by beads in Homogenizing buffer (10 mM HEPES (pH 7.4), 0.25 M sucrose, protease inhibitor, 50% Glo-Lysis buffer) at 4 °C. Trichloroacetic acid (final concentration 0.8%) was added to homogenized sample, and the homogenate was centrifuged at 12,000 rpm at 4 °C for 15 min. Supernatants were neutralized with 1 M HEPES (pH 8.0). Chemiluminescence of the reaction mixtures was measured with an ARVO multi-label counter (Perkin Elmer, Inc.).

### 2.7 Extracellular Flux Analyses

Extracellular acidification rate (ECAR) and oxygen consumption rate (OCR) were determined in collagen-coated XF96-well plates using an XF96 extracellular flux analyzer (Seahorse Bioscience, Billerica, MA, USA). Briefly, 0.8 × 10^4^ PC12 cells were cultured for 24 h in XF96-well plates in DMEM supplemented with 50 ng/ml NGF, 1% FBS, and 1% HS. Subsequent esculetin treatment was for 24 h. On the day of analysis, cells were washed once with assay medium (Seahorse Bioscience) (unbuffered DMEM with initial pH 7.4, based on the manufacturer's protocol, plus 5.6 mM glucose (Nacalai Tesque) and 1 mM Na pyruvate (Nacalai Tesque)), and then incubated with fresh assay medium for 1 h in a 37 °C non-CO_2_ incubator. ECAR and OCR were monitored using a Seahorse Bioscience XF96 extracellular flux analyzer. ECAR and OCR rates at each time point were averaged from 5 replicate wells (ECAR) and 7 replicate wells (OCR). ECAR and OCR were determined for each well and were normalized by cell number. The mitochondrial complex I inhibitor rotenone (3 μM) (Sigma), the mitochondrial complex III inhibitor antimycin (3 μM) (Sigma), the mitochondrial complex V inhibitor oligomycin (3 μM) (Sigma), the pharmaceutical uncoupler CCCP (1 μM) (Nacalai Tesque), and 2-deoxy-d-glucose (50 mM) (Nacalai Tesque) were used as inhibitors.

### 2.8 RT-qPCR

Total RNA was purified from cultured PC12 cells using an SV total RNA isolation kit (Promega), following the manufacturer's protocol. For cDNA synthesis, total RNA (1 μg) was reverse transcribed using ReverTra Ace qPCR RT Master Mix with gDNA Remover (TOYOBO, Osaka, Japan). qPCR was performed on a Light Cycler System (Roche Diagnostics GmbH, Mannheim, Germany), with the following program: 98 °C for an initial 2 min followed by 45 cycles of denaturation at 98 °C for 10 s, annealing at 60 °C for 10 s, and extension at 68 °C for 30 s, using KOD SYBR qPCR Mix (TOYOBO). The PCR primers were the following:

rat GAPDH/F: 5′- ATGTATCCGTTGTGGATCTGAC -3′
rat GAPDH/R: 5′- CCTGCTTCACCACCTTCTTG -3′
rat ATF4/F: 5′- TAGAGCTGGGAAGTGAGGTTGA -3′
rat ATF4/R: 5′- AGTGTCTTCCTCCTTTACACACTG -3′
rat CHOP/F: 5′- CAGGAGGTCCTGTCCTCAGA -3′
rat CHOP/R: 5′- GGGATGCAGGGTCAAGAGTAG -3′

### 2.9 Western Blot Analysis

Cells were collected and lysed in RIPA buffer (50 mM Tris-HCl pH 7.6, 150 mM NaCl, 1% NP40, 0.5% sodium deoxycholate, 0.1% SDS (all purchased from Nacalai Tesque)) containing 1 mM NaF, 1 mM sodium disphosphate decahydrate, 1 mM NaVO_4_, 0.5 mM PMSF, 1 × protease inhibitor cocktail (all purchased from Nacalai Tesque), 0.1% CHAPS (Dojindo, Kumamoto, Japan), and 10 mM β-glycerophosphate (Sigma). Dissected brain sample was weighed and homogenized by beads and a homogenizer in ice-cold buffer (50 mM Tris-HCl pH 7.6, 150 mM NaCl, 1% NP40, 0.5% sodium deoxycholate, 0.1% SDS, 0.1% CHAPS) containing protease inhibitors and phosphatase inhibitors. Samples were sonicated on ice and centrifuged at 4 °C at 15,000 rpm for 15 min, and the supernatant was collected. Proteins (15–20 μg per lane) were loaded, and were separated by 10%, 12%, or 15% SDS-PAGE and transferred to polyvinylidene fluoride membranes (Millipore, Billerica, MA, USA). Primary antibodies were as follows: anti-actin (1:2000, Millipore, MAB1501), anti-CHOP (1:400, Santa Cruz Biotechnology, Dallas, TX, USA, sc-793), anti-AMPK (1:1000, Cell Signaling Technology, Danvers, MA, USA, #2603S), anti-Phospho-AMPK (Thr172) (1:1000, Cell Signaling Technology, #2535S), anti-Parkin (1:1000, Abcam, Cambridge, MA, USA, ab179812), anti-PINK1 (1:1000, Abcam, ab23707), anti-LC3 (1:1000, Cell Signaling Technology, #2775), anti-p62 (1:1000, Medical & Biological Laboratories, Aichi, Japan, PM045), anti-Mitofusin2 (1:1000, Abcam, ab56889), anti-Drp1 (1:1000, Abcam, ab184247), anti-Bax (1:100, Santa Cruz Biotechnology, sc-493), anti-caspase3 (1:1000, Cell Signaling Technology, #9662), anti-Tyrosine hydroxylase (1:1000, Millipore, AB152). HRP-labeled secondary antibodies (GE Healthcare UK Ltd., Buckinghamshire, England) were used for visualization by enhanced chemiluminescence (GE Healthcare).

### 2.10 Imaging of ATP Concentrations in PC12 Cells

We first established PC12 cells continuously expressing cyt-ATeam (AT1.03^εD12N/εD109G^) or mit-ATeam (Imamura et al., 2009). In imaging, the cells were plated on a collagen-coated glass-bottom 35 mm dish (glass thickness 0.15–0.18 mm, Mat-tek, Ashland, MA, USA), and were cultured in phenol red-free DMEM (Nacalai Tesque). Wide-field observations of the cells were performed on a Nikon Ti-E inverted microscope using a 40 × objective (Nikon, Tokyo, Japan; CFI Plan Apo λ 40 × dry: NA 0.95) for cyt-ATeam, and 60 × objective (Nikon, Tokyo, Japan; CFI Plan Apo λ 60 × oil: NA 1.40) for mit-ATeam, and the following filter sets (Semrock, Rochester, NY, USA): for dual emission ratio imaging of cyt-ATeam and mit-ATeam, 438/24 excitation filter - dichroic mirror FF458-483/32 emission filter for CFP and 542/27 emission filter for YFP. Cells were illuminated using a xenon lamp through 12.5 and 25% neutral density filters. Fluorescence emissions from ATeam biosensors were captured with a Zyla4.2 sCMOS camera (ANDOR, Oxford instruments, USA). The exposure times were 200 ms for CFP and YFP images. The microscope system was controlled with NIS-Elements (Nikon). Cells were maintained on the microscope at 37 °C with a continuous supply of a mixture of 95% air and 5% carbon dioxide using a stage-top incubator (Tokai Hit, Shizuoka, Japan). Imaging data were analyzed using MetaMorph (Molecular Devices, Sunnyvale, CA, USA). The YFP/CFP emission ratio was calculated by dividing YFP intensity by CFP intensity for each cell.

### 2.11 Transmission Electron Microscope (TEM)

Cultured cells were fixed in 4% paraformaldehyde with 2% glutaraldehyde overnight at 4 °C. Sections were exposed to 1% osmium tetroxide in 0.1 M phosphate buffer (pH 7.4), for 60 min at room temperature, then dehydrated in a series of graded ethanol solutions, and embedded in epoxy-resin Luveak812 (Nacalai Tesque) according to the inverted beam capsule procedure and polymerized at 60 °C for 3 days. Ultrathin sections were prepared on an ultramicrotome (EM UC6; Leica, Heidelberg, Germany), and stained with uranyl acetate and lead citrate, and then were observed with a Hitachi H-7650 transmission electron microscope (Hitachi, Tokyo, Japan).

### 2.12 Measurement of Mitochondrial Length

For imaging, the cells were plated on a collagen-coated glass-bottom 35 mm dish (glass thickness 0.15–0.18 mm, Mat-tek). For measurement of mitochondrial length, the cells were cultured in phenol red-free DMEM. Mitochondria were labeled with 50 nM of MitoTracker Green FM (MTG; Invitrogen, Carlsbad, CA, USA), and nuclear DNA was labeled with Hoechst33342 (Hoechst; Invitrogen). Wide field observations of the cells were performed on a Nikon Ti-E inverted microscope using a 60 × objective (Nikon; CFI Plan Apo λ 60 × oil: NA 1.40) and the following filter sets (Semrock): for imaging of MTG, 482/18 excitation filter - dichroic mirror FF495-520/35 emission filter; and for imaging of Hoechst, 405/10 excitation filter - dichroic mirror FF495-520/35 emission filter. The exposure times were 200 ms for MTG and 400 ms for Hoechst. The microscope system was controlled with NIS-Elements (Nikon). Cells were illuminated using a xenon lamp through 12.5 and 25% neutral density filters. Fluorescence emissions from cells were captured with a Zyla4.2 sCMOS camera (ANDOR). Cells were maintained on the microscope at 37 °C with a continuous supply of a mixture of 95% air and 5% carbon dioxide using a stage-top incubator (Tokai Hit).

### 2.13 Primary Mesencephalic Dopaminergic Cultures

Mesencephalic tissue (ventral midbrain) was dissected from embryonic day (E) 14 mice, tissue pieces were collected in Dissection buffer (HBSS, 2% penicillin/streptomycin (P/S; Nacalai Tesque), 20 mM d-glucose, 200 μM ascorbic acid (Nacalai Tesque)), transferred to 1 mg/ml papain (Nacalai Tesque) solution containing 12.5 μg/ml DNase I (Worthington biochemical corporation) and 5 mM MgCl_2_-6H_2_O, and incubated at 37 °C for 15 min. Then, tissue pieces were homogenized in Differentiation medium (Neurobasal Medium (Gibco), 2% B-27 supplement (Gibco), 1 × GlutaMax (Gibco), 1% FBS, 1% P/S, 200 μM ascorbic acid) containing 12.5 μg/ml DNase I and 5 mM MgCl_2_-6H_2_O, and centrifuged at 200 ×* g*, resuspended in Differentiation medium and plated on poly-d-lysine coated glass plates in 24-well plates (Gaven et al., 2014, Iwakura et al., 2011). After 7 days in vitro (DIV), cultured neurons were treated with 25 μM KUSs or 25 μM esculetin for 24 h, and then further incubated with 10 μM MPP + for 48 h. Subsequently, cells were fixed with 4% paraformaldehyde in PBS at room temperature for 30 min, washed with PBS, and permeabilized in 0.5% Triton X-100 (Nacalai Tesque)/5% goat serum at room temperature for 30 min. Then, cultures were treated with primary antibodies at 4 °C overnight, and with secondary antibodies at room temperature for 6 h. The stereomorphological analyses were performed on dopaminergic neurons by BZ-X700 fluorescence microscope (KEYENCE, Osaka, Japan) with its analysis application programs. Primary antibodies used for immunofluorescence staining were anti-tyrosine hydroxylase (1:1000, Millipore, AB152), anti-NeuN antibody (1:500, Millipore, MAB377). The secondary antibodies were Alexa488-conjugated anti-rabbit (1:200, Invitrogen), and Alexa546-conjugated anti-mouse (1:200, Invitrogen). In order to determine dopaminergic neuron viability following MPP + treatment, both in cell bodies and neurites, mesencephalic cultures were processed for TH immunoreactivity and counted using a BZ-X700 fluorescence microscope (KEYENCE).

### 2.14 MPTP Parkinson's Disease Model Mice

KUS121, esculetin, GSK4716 (G47), or GSK5182 (G51) was dissolved in 5% gamma-cyclodextrin (γ-CD) (Tokyo Chemical Inc., Tokyo, Japan) to make a 5 mg/ml solution, and was sonicated for 6 h at 4 °C. In the MPTP Parkinson's mouse model, oral administration of KUS121 (50 mg/kg/day), esculetin (50 mg/kg/day), G47 (2.5 mg/kg/day), or G51 (5 mg/kg/day) was performed 4 days per week, starting 1 week before probenecid and MPTP injection. 6 week-old male C57BL/6N Slc mice weighing 20–25 g were used for the chronic MPTP Parkinson's disease model. Animals were housed at a controlled room temperature (25 ± 1 °C) and humidity (54 ± 1%) under a 12-hour light/12-hour dark cycle. Animals were allowed access to food and water ad libitum. Animals were randomly divided into ten groups: no drug-treated (NT) group (*n* = 7); KUS121 group (*n* = 6); esculetin group (*n* = 6); G47 group (*n* = 5); G51 group (*n* = 5); MPTP group (*n* = 7); MPTP + KUS121 group (*n* = 7); MPTP + esculetin group (*n* = 7); MPTP + G47 group (*n* = 7); and MPTP + G51 group (*n* = 7). The mice were injected intraperitoneally with 20 mg/kg of MPTP hydrochloride (Sigma) in combination with probenecid (250 mg/kg in PBS, i.p.), over 5 months at 3.5 day intervals.

### 2.15 Rotarod Test

The rotarod test was performed to measure forelimb and hindlimb motor coordination and balance. Mice were placed on the rotating rod using the following steps: for 1 week before MPTP injection, mice were trained to stay on the rotarod at constant speed (16 rpm) for 240 s. For each trial, the duration until the mice fell off the rotating rod (constant speed, 16 rpm) was recorded. Mice received three trials in one day without doing consecutive trials. Mean latencies and standard deviations were calculated and used for analysis.

### 2.16 Histochemical Analyses

Mouse brains were fixed with 4% paraformaldehyde and 1% glutaraldehyde overnight at 4 °C and embedded in paraffin, and coronal sections were prepared with a thickness of 3 μm. The sections were treated with primary antibodies at room temperature overnight, and with secondary antibodies at room temperature for 2 h. In order to enhance immune signals, a Vectastain elite ABC standard kit (Vector Laboratories, Burlingame, CA, USA) was used, following the manufacturer's protocol. The stereomorphological analyses were performed on the substantia nigra of the mouse brain with a BZ-9000 Generation II microscopy and a BZ-X700 fluorescence microscope (KEYENCE), along with its analysis application programs. The primary antibodies used for immunocytochemistry were anti-tyrosine hydroxylase (1:2000, Millipore, AB152) and anti-α-synuclein (1:50, BD Transduction Laboratories, Lexington, KY, 610787). The secondary antibodies were biotinylated anti-rabbit IgG (H + L) (Vector Laboratories) and biotinylated anti-mouse IgG (H + L) (Vector Laboratories). Primary antibodies used for immunofluorescence staining were anti-tyrosine hydroxylase (1:1000, Millipore), anti-CHOP (1:100, Cell Signaling Technology, L63F7), and anti-α-synuclein (1:50, BD Transduction Laboratories). The secondary antibodies were Alexa488-conjugated anti-rabbit (1:200, Invitrogen), and Alexa546-conjugated anti-mouse (1:200, Invitrogen). For immunofluorescence, stained brain sections were counterstained using VECTASHIELD Mounting Medium with DAPI (Vector Laboratories).

### 2.17 Imaging of ATP Concentrations in the Brain Sections of Go-ATeam2 Mice

Go-ATeam2 mice express a FRET-based ATP biosensor similar to the one used for cell culture, except the fluorescent protein reporters are OFP and GFP, rather than YFP and CFP, respectively (M.Y. et al., manuscript in preparation). Go-ATeam2 mice were anesthetized with 4% isoflurane inhalation with oxygen and the mouse brains were immediately dissected. Collected brains were immersed in Ringer's solution (119 mM NaCl, 2.5 mM KCl, 1 mM NaH_2_PO_4_·2H_2_O, 26.2 mM NaHCO_3_, 11 mM d-glucose, 2.5 mM CaCl_2_, 1.3 mM MgSO_4_) gassed with 95% O_2_ and 5% CO_2_ as soon as possible. During production of 300 μm brain slices with a vibratome (Leica Microsystems GmbH, Germany), the brain was kept in ice-cold Ringer's solution (gassed with 95% O_2_ and 5% CO_2_). In order to allow the slices to recover from the cutting procedure, slices were incubated at room temperature for approximately 1 h in Ringer's solution (gassed with 95% O_2_ and 5% CO_2_). Observations of brain sections were performed on a Leica M165 FC stereo microscope (Leica) using a 1 × objective (Leica, Plan Apo 1.0) and the following DualView2 filter sets (INDEC Biosystems, Santa Clara, CA, USA): for dual emission ratio imaging of Go-ATeam2, 470/40 excitation filter - dichroic mirror 540 DCLP - 515/30 for GFP and 575/40 for OFP. Fluorescence emission from Go-ATeam2 was captured using ORCA-Flash4.0 (Hamamatsu, Japan). The exposure times were 4 s for GFP and OFP images. Imaging data of OFP/GFP emission ratios were analyzed using MetaMorph (Molecular Devices, Sunnyvale, CA).

### 2.18 Statistical Analysis

For statistical analyses in experiments using cells (PC12 cells, HEK293A cells) or mice treated with or without KUSs or esculetin, ANOVA with Tukey (if variances were not unequal), Games-Howell (if variances were unequal) post-hoc test, or Dunnett's test were used. Statistical analyses were performed using PASW Statistics version 17.0 (SPSS Inc., Chicago, IL, USA). The level of statistical significance was set at *P* < 0.05. Specific statistical tests are indicated in figure legends.

## 3 Results

### 3.1 Identification of Esculetin as an ERR Agonist, and Its Ability to Enhance ATP Production

**Fig. 1:**
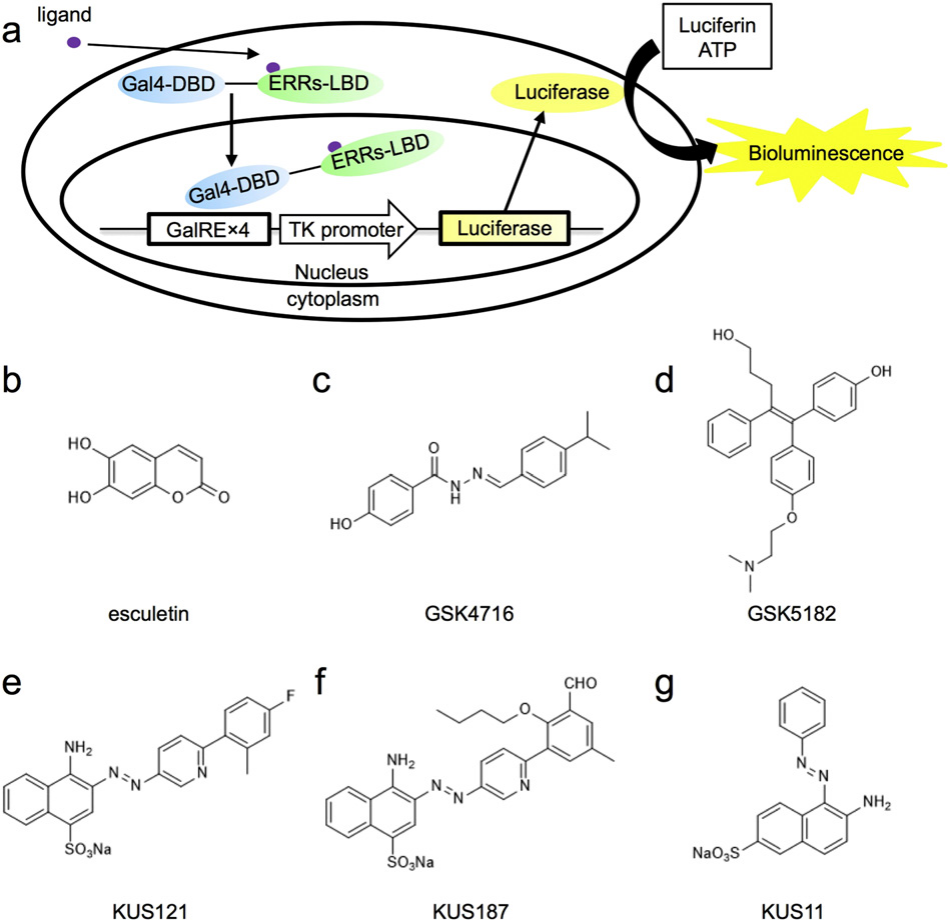
Schematic drawing of the luciferase-based reporter assay and chemical structures of compounds used in this study. (a) Schematic drawing of the luciferase-based estrogen receptor-related receptors (ERRs) reporter assay. (b–g) Structures of esculetin (b), GSK4716 (c), GSK5182 (d), KUS121 (e), KUS187 (f), and KUS11 (g) (Ikeda et al., 2014).

In order to search for ERR agonists, we used our previous assay system for nuclear receptor transactivation (Kamei et al., 2003) (Fig. 1a). In this system, ligand binding domains of human ERRs were fused with the Gal4 DNA binding domain, and each of the resulting Gal4-ERR fusion proteins was expressed in HEK293A cells, together with a luciferase reporter plasmid, which contains 4 copies of GalRE (Kamei et al., 2003, Yamamoto et al., 2001), a Gal4-responsive element in the promoter region. We also co-transfected a plasmid expressing β-galactosidase for the normalization of transfection efficiencies. Twenty-four hours after the transfection, measurable levels of both luciferase and β-galactosidase were observed.

**Fig. 2:**
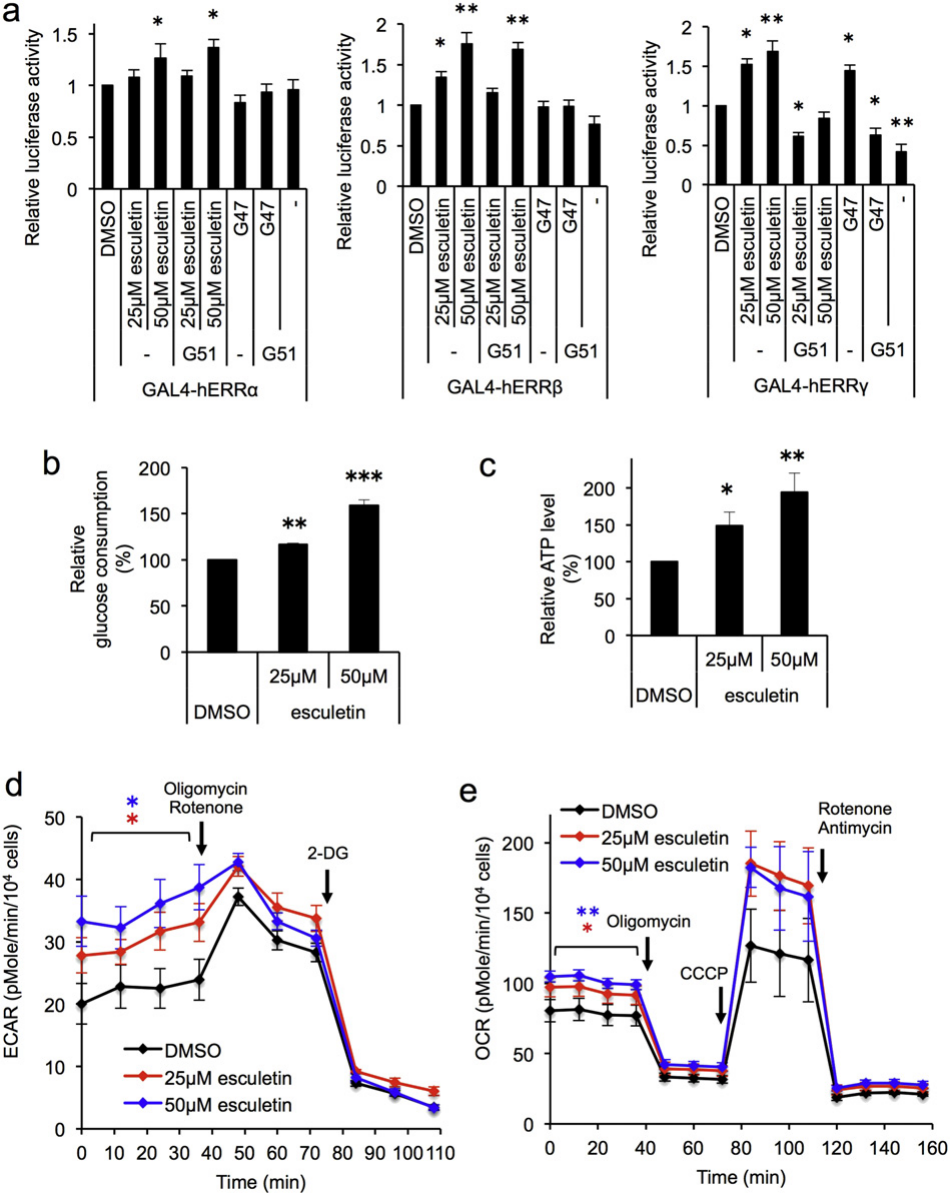
Identification of esculetin as an ERR agonist, and its ability to enhance ATP production. (a) Transcriptional activation profiles of esculetin via ERRα, β, γ. See details in “Materials and Methods”. Mean values of relative luciferase activities, after normalization with β-galactosidase activities, are shown, with values for DMSO alone set at 1.0. Error bars indicate standard deviations. *P < 0.05, **P < 0.01, ANOVA with Games-Howell post-hoc test (*n* = 4), vs. DMSO. (b) Effects of esculetin on glucose consumption. In the absence (DMSO) or the presence of 25 μM or 50 μM esculetin, neuronally differentiated PC12 cells were incubated for 24 h, then total amounts of glucose consumption and live cell numbers were measured. Mean values of relative glucose consumption per cell were calculated and are shown, with values for DMSO alone set at 100%. Error bars indicate standard deviations. **P < 0.01, ***P < 0.001, ANOVA with Tukey's post-hoc test (*n* = 3), vs. DMSO. (c) Effects of esculetin on intracellular ATP levels. Neuronally differentiated PC12 cells were cultured with the conditions shown in (b), and then total ATP amounts and live cell numbers were determined. Mean values of relative ATP levels per cell were calculated and are shown, with values for DMSO alone set at 100%. Error bars indicate standard deviations. *P < 0.05, **P < 0.01, ANOVA with Games-Howell post-hoc test (*n* = 4), vs. DMSO. (d, e) Effects of esculetin on energy flux. Neuronally differentiated PC12 cells were incubated for 24 h in the absence (DMSO) or the presence of 25 μM or 50 μM esculetin, and then the extracellular acidification rate (ECAR) and oxygen consumption rate (OCR) were measured. Error bars indicate standard deviations (ECAR; *n* = 5, OCR; *n* = 7). Other chemicals used in this experiment were the following: 3 μM rotenone, 3 μM antimycin, 3 μM oligomycin, 1 μM CCCP, and 50 mM 2-deoxy-d-glucose (2-DG). Quantified data are presented in Fig. S1. *P < 0.05, **P < 0.01, ANOVA with Games-Howell post-hoc test, vs. DMSO.

Using this assay system, we screened a chemical library (approximately 100,000 chemicals) and noted that esculetin (CAS No. 305-01-1) (Fig. 1b), apart from 47 pseudo-positives, was able to specifically transactivate the luciferase reporters through all of the Gal4-ERRs, most strongly through Gal4-ERRγ, followed by Gal4-ERRβ, and weakly by Gal4-ERRα (Fig. 2a). The Gal4-ERRγ-mediated transactivation by esculetin or GSK4716 (CAS No. 101574-65-6) (Fig. 1c), a known ERRγ agonist, was cancelled by the addition of GSK5182 (CAS No. 877387-37-6) (Fig. 1d), an ERRγ-specific inverse-agonist (Fig. 2a). Consistently, the Gal4-ERRα- or Gal4-ERRβ-mediated transactivation was not cancelled by the addition of GSK5182 (Fig. 2a). These results indicated that esculetin is able to function as an ERR agonist for all three ERRs.

Given that esculetin functions as an ERR agonist, it was expected that esculetin can alter the metabolic state of cells. We therefore examined this possibility. As expected, addition of esculetin dose-dependently enhanced glucose uptake as well as cellular ATP levels (Fig. 2b, c). Consistently, flux analyses revealed that addition of esculetin stimulated both glycolysis and oxygen consumption, also apparently in a dose-dependent manner (Fig. 2d, e, Fig. S1a, b). Note that treatment with CCCP (Carbonyl cyanide *m*-chlorophenyl hydrazine), a respiratory chain uncoupler, revealed that esculetin treatment raised not only basal respiration but also spare respiratory capacities and maximal respiration (Fig. 2e, Fig. S1).

### 3.2 KUSs and Esculetin Prevent MPP +-induced ATP Depletion, ER Stress, and Cell Death in PC12 Cells

We previously showed that KUSs, e.g. KUS121 (CAS No. 1357164-52-3) (Fig. 1e) and KUS187 (CAS No. 1357164-95-4) (Fig. 1f), were able to prevent ATP depletion, ER stress, and cell death in neuronally differentiated PC12 cells treated with antimycin and oligomycin, which are specific inhibitors of mitochondrial respiratory chain complex III and V, respectively (Ikeda et al., 2014, Nakano et al., 2016). We then examined whether KUS121 and esculetin could show similar effects in PC12 cells treated with several other types of mitochondrial respiratory chain inhibitors. In addition to previously tested antimycin and oligomycin, we used MPP +, rotenone, and metformin, all of which are inhibitors of mitochondrial respiratory chain complex I.

**Fig. 3:**
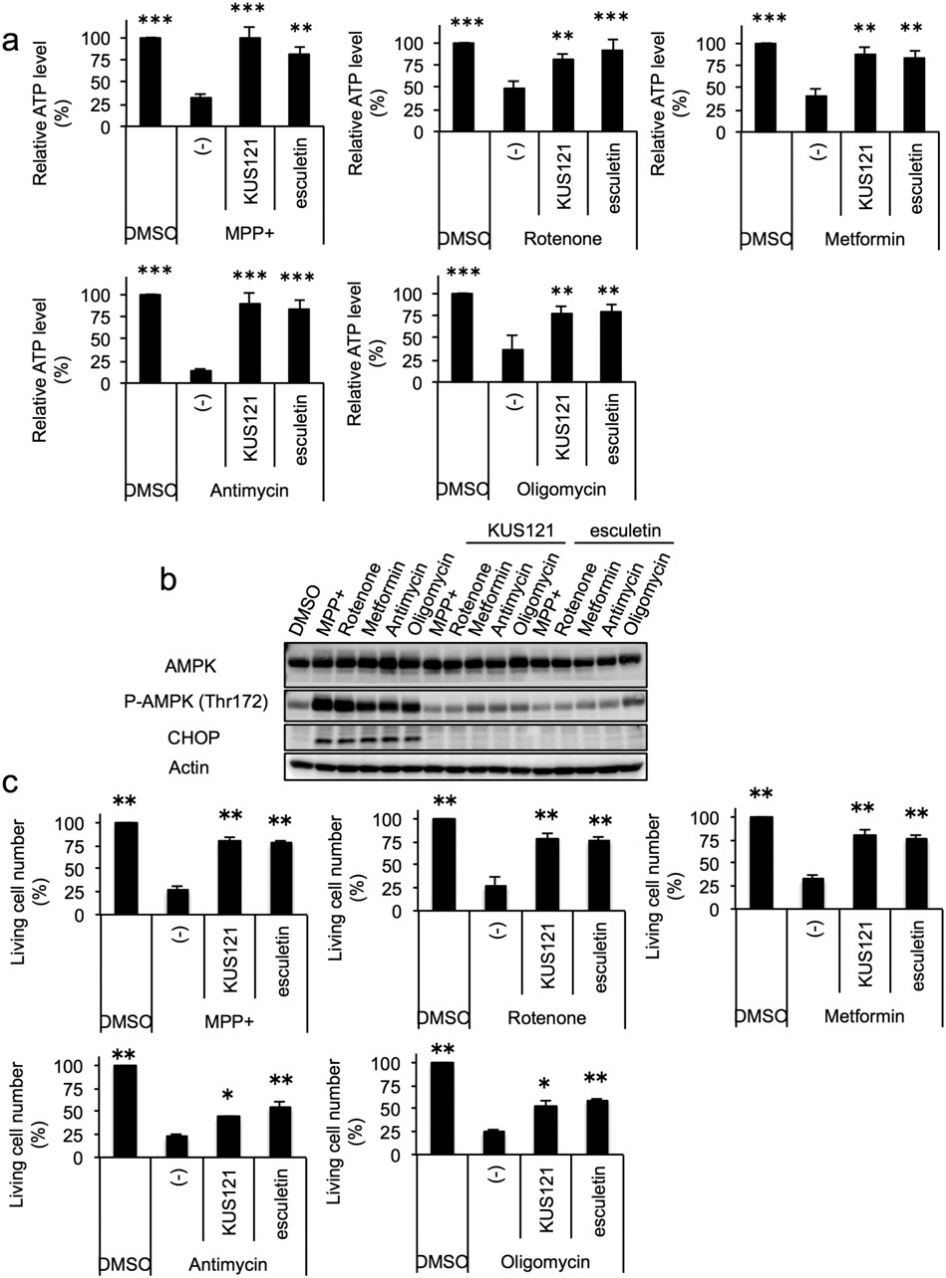
KUS121 and esculetin prevent mitochondrial dysfunction-induced ATP decrease, ER stress, and cell death in neuronally differentiated PC12 cells. (a) Effects of KUS121 and esculetin on the prevention of ATP decreases induced by mitochondrial respiratory chain complex inhibitors. Neuronally differentiated PC12 cells were incubated for 24 h in the absence (DMSO) or the presence of 50 μM KUS121 or 50 μM esculetin, and then further incubated with each of the mitochondrial respiratory chain complex inhibitors (75 μM MPP +; 10 nM rotenone; 3 mM metformin; 100 nM antimycin; 0.01 μg/ml oligomycin) for 24 h. Then, total ATP amounts and live cell numbers were determined. Mean values of relative ATP levels per cell were calculated and are shown, with values for DMSO alone set at 100%. Error bars indicate standard deviations. **P < 0.01, ***P < 0.001, ANOVA with Tukey's post-hoc test (*n* = 4), vs. MPP +, Rotenone, Metformin, Antimycin, or Oligomycin alone (-). (b) Effects of KUS121 and esculetin on the prevention of AMPK phosphorylation and ER stress induced by mitochondrial respiratory chain complex inhibitors. Neuronally differentiated PC12 cells were cultured with the conditions shown in (a), and were subjected to western blot analyses. Actin served as a loading control. (c) Effects of KUS121 and esculetin on the prevention of cell death induced by mitochondrial respiratory chain complex inhibitors. Neuronally differentiated PC12 cells were incubated for 24 h in the absence (DMSO) or the presence of 50 μM KUS121 or 50 μM esculetin, and then further incubated with each of the mitochondrial respiratory chain complex inhibitors shown in (a) for 28 h. Then, live cell numbers were counted by staining with trypan blue. Mean values of relative live cell numbers are shown, with values for DMSO alone set at 100%. Error bars indicate standard deviations. *P < 0.05, **P < 0.01, ANOVA with Games-Howell post-hoc test (*n* = 3), vs. MPP +, Rotenone, Metformin, Antimycin, or Oligomycin alone (-).

We treated neuronally differentiated PC12 cells with MPP +, rotenone, metformin, antimycin, and oligomycin in the presence or the absence of KUS121 or esculetin for 24 h; cellular ATP levels were then measured by the luciferase-based assay. All of the inhibitors clearly lowered ATP levels, and KUS121 and esculetin nearly equally suppressed this reduction in ATP levels (Fig. 3a). Under these conditions, KUS121 and esculetin inhibited AMPK activation (evidenced by the phosphorylation of AMPK Thr172), as well as CHOP induction, an ER stress marker with cell death-inducing activity (Zinszner et al., 1998) (Fig. 3b). Consistent with these results, KUS121 and esculetin were able to suppress cell death by these mitochondrial respiratory chain inhibitors (Fig. 3c, Fig. S2). Interestingly, KUS121 and esculetin appeared to more strongly prevent cell death induced by MPP +, rotenone, and metformin, which are inhibitors of mitochondrial respiratory chain complex I, than antimycin and oligomycin, which are inhibitors of mitochondrial respiratory chain complex III and V, respectively (Fig. 3c, Fig. S2).

**Fig. 4:**
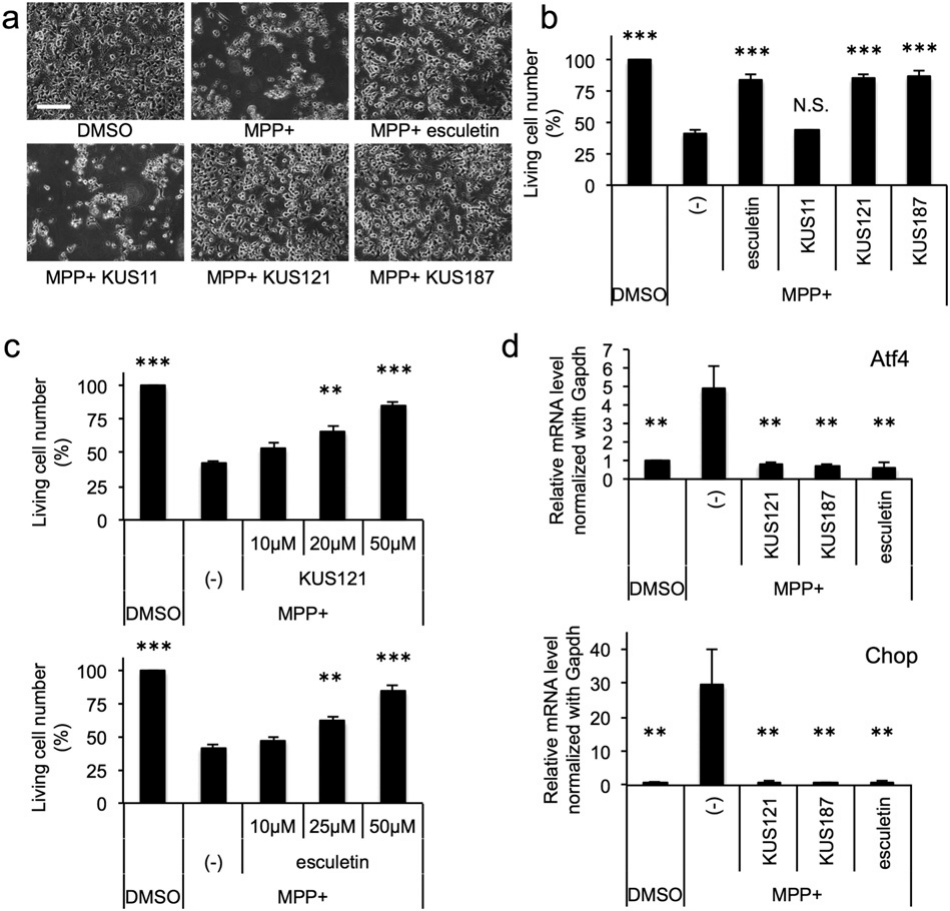
Efficacies of KUSs and esculetin in the MPP +-induced Parkinson's disease cell culture model. (a–c) Effects of KUSs (KUS121 and KUS187) and esculetin on the prevention of cell death induced by MPP +. Neuronally differentiated PC12 cells were incubated for 24 h in the absence (DMSO) or the presence of KUSs (50 μM in (a, b); 10, 20, or 50 μM in (c)) or esculetin (50 μM in (a, b); 10, 25, or 50 μM in (c)), and then further incubated with 75 μM MPP + for 28 h. Then, cells were photographed (a) and live cell numbers were counted by staining with trypan blue (b, c). Mean values of relative live cell numbers are shown, with values for DMSO alone set at 100%. Error bars indicate standard deviations. Scale bar, 200 μm. (b) N.S., not significant, ***P < 0.001, Dunnett's test (*n* = 3), vs. MPP + alone (-) (c) **P < 0.01, ***P < 0.001, ANOVA with Tukey's post-hoc test (*n* = 3), vs. MPP + alone (-). A time course of the effects of KUSs and esculetin on the prevention of cell death of neuronally differentiated PC12 cells treated with MPP + is shown in Fig. S17. (d) Effects of KUSs and esculetin on the prevention of ER stress induced by MPP +. Neuronally differentiated PC12 cells were incubated for 24 h in the absence (DMSO) or the presence of 50 μM KUSs or 50 μM esculetin, and then further incubated with 75 μM MPP + for 24 h. Then, mRNA levels of ATF4 and CHOP were analyzed by RT-qPCR. GAPDH mRNA served as an internal control for RT-qPCR. Mean values of relative mRNA levels are shown, with values for DMSO alone set at 1.0. Error bars indicate standard deviations. **P < 0.01, ANOVA with Tukey's post-hoc test (*n* = 3), vs. MPP + alone (-). A time course of the effects of KUSs and esculetin on the phosphorylation of AMPK, CHOP induction, and caspase activation are shown in Fig. S18.

In order to examine the potential benefits of KUSs and esculetin for Parkinson's disease in more detail, we analyzed the neuroprotective efficacies of these compounds with MPP + exposure. Neuronally differentiated PC12 cells were treated with 75 μM MPP + for 28 h, and then live cell (or dead cell) numbers were estimated (Fig. 4a, b, Fig. S3). With this treatment, approximately 60% of the cells died. This cell death was not inhibited by KUS11 (CAS No. 146293-60-9) (Fig. 1g), which has no inhibitory activity on VCP ATPase (Ikeda et al., 2014). By contrast, addition of KUS121, KUS187, and esculetin significantly protected the cells from cell death; on average, > 80% cells survived after MPP + treatment (Fig. 4b). For KUS121 and esculetin, we further confirmed that the rescue was dose-dependent with these compounds (Fig. 4c, Fig. S3). Consistent with these results, MPP + treatment induced ATF4 and CHOP mRNAs, and addition of KUS121, KUS187, or esculetin completely inhibited these inductions (Fig. 4d). We confirmed the ATP-maintaining effects, the inhibitory effects on ER stress and on AMPK activation, and the neuroprotective effects of KUSs and esculetin in MPP +-treated fully differentiated PC12 cells (Fig. S4). It is also notable that the effects of KUS121 and esculetin were additive with regard to the prevention of all of the MPP +-induced features-ATP decrease, AMPK activation, CHOP induction (ER stress), and cell death-at doses of 25 μM each (Fig. S5).

We also examined the effects of KUSs and esculetin on cellular calcium dynamics. KUS121, KUS187, or esculetin alone did not affect calcium levels in neuronally differentiated PC12 cells (Fig. S6). Addition of MPP + alone did not either. However, addition of both MPP + and esculetin, but not MPP + and KUSs, significantly reduced cellular calcium levels (Fig. S6). These results suggest that calcium levels are not a critical determinant of MPP +-induced cell death.

**Fig. 5:**
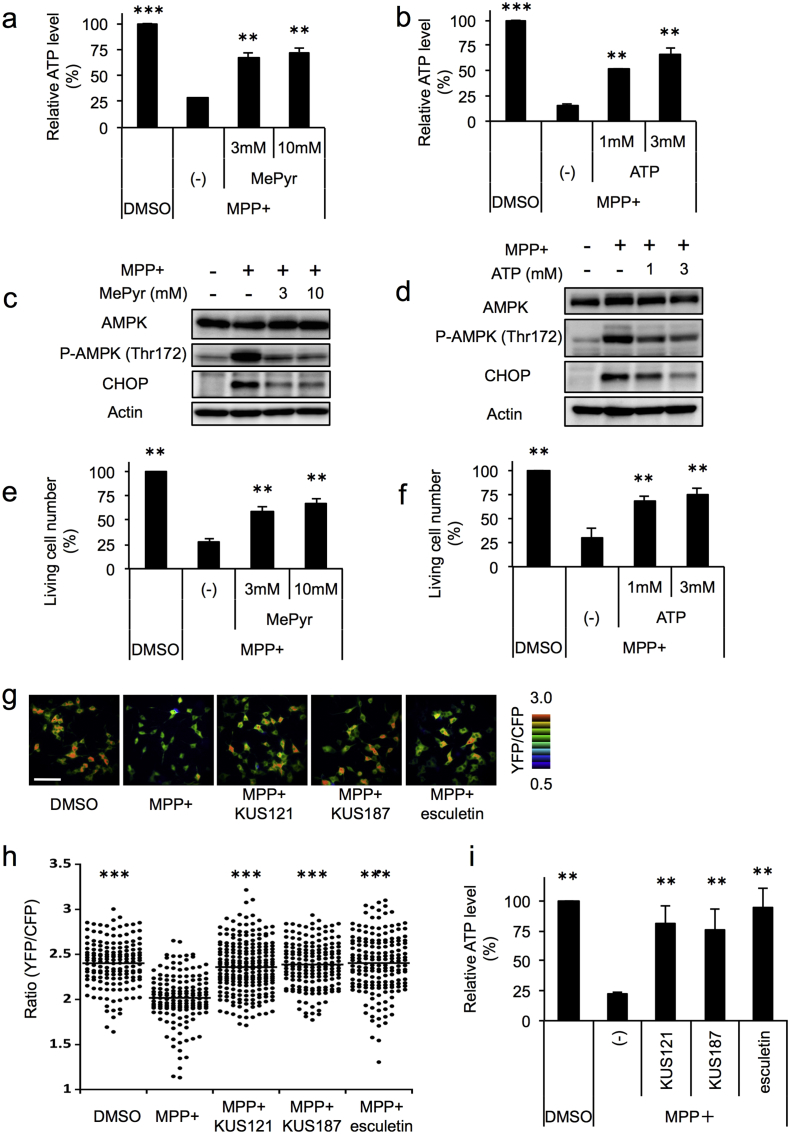
Efficacies of maintaining ATP levels by methylpyruvate or ATP treatment in the MPP +-induced Parkinson's disease cell culture model. (a, b) Neuronally differentiated PC12 cells were treated with 75 μM MPP + for 19 h, then methylpyruvate (MePyr) (3 and 10 mM) or ATP (1 and 3 mM) was added, and cells were incubated for another 5 h. Then, total ATP amounts and live cell numbers were determined. Mean values of relative ATP levels per cell were calculated and are shown, with values for DMSO alone set at 100%. Error bars indicate standard deviations. **P < 0.01, ***P < 0.001, ANOVA with Games-Howell post-hoc test (*n* = 3 or 4), vs. MPP + alone (-). (c, d) Effects of MePyr or ATP treatment on the prevention of ER stress induced by MPP +. Neuronally differentiated PC12 cells were cultured with the conditions shown in (a, b), and were subjected to western blot analyses. Actin served as a loading control. (e, f) Effects of MePyr or ATP treatment on the prevention of cell death induced by MPP +. Neuronally differentiated PC12 cells were cultured with the conditions shown in (a, b) for 28 h. Then, live cell numbers were counted by staining with trypan blue. Mean values of relative live cell numbers are shown, with values for DMSO alone set at 100%. Error bars indicate standard deviations. **P < 0.01, ANOVA with Games-Howell post-hoc test (*n* = 3), vs. MPP + alone (-). (g) Representative ratiometric pseudocolor FRET images of cyt-ATeam1.03-expressing PC12 cells. Neuronally differentiated PC12 cells expressing cyt-ATeam1.03 were incubated for 24 h in the absence (DMSO) or the presence of 50 μM KUSs or 50 μM esculetin, and then further incubated with 75 μM MPP + for 24 h. The YFP/CFP emission ratio was calculated by dividing YFP intensity by CFP intensity for each cell. Scale bar, 200 μm. (h) Distributions of YFP/CFP emission ratios of cyt-ATeam1.03. The ratios were calculated from the fluorescent images in (g). Images of cells were analyzed for each condition, as follows: DMSO, 154 cells; MPP +, 150 cells; MPP + KUS121, 223 cells; MPP + KUS187, 166 cells; MPP + esculetin, 176 cells. ***P < 0.001, ANOVA with Games-Howell post-hoc test, vs. MPP +. (i) Measurements of the relative ATP levels per cell. Neuronally differentiated PC12 cells were cultured with the same conditions as in (g). Then, total ATP amounts and live cells numbers were determined. Mean values of relative ATP levels per cell were calculated and are shown, with values for DMSO alone set at 100%. Error bars indicate standard deviations. **P < 0.01, ANOVA with Tukey's post-hoc test (*n* = 3), vs. MPP + alone (-).

We have shown that addition of methylpyruvate (membrane-permeable pyruvate, which is converted to ATP in mitochondria) as well as ATP itself has the ability to reduce tunicamycin-induced ER stress and ATP decrease (Ikeda et al., 2014). Consistent with these results, addition of 3 mM or 10 mM methylpyruvate was able to prevent the MPP +-induced ATP decrease, CHOP expression, and cell death (Fig. 5a, c, e, Fig. S7a). Likewise, addition of 1 mM or 3 mM ATP was able to prevent the MPP +-induced ATP decrease, CHOP expression, and cell death (Fig. 5b, d, f, Fig. S7b). Prevention of the MPP +-induced decrease of ATP levels by methylpyruvate or ATP was further evidenced by decreased levels of phosphorylated AMPK (Fig. 5c, d). These results demonstrated that ATP levels are a crucial determinant of ER stress and cell death.

Recently, we have developed several different FRET probes for ATP (ATeams), which allow us to estimate relative ATP levels in live cells (Imamura et al., 2009, Nakano et al., 2011, Tsuyama et al., 2013). In order to examine whether an ATeam probe could detect changes of ATP levels in the cytoplasm of MPP +-treated live PC12 cells, we created PC12 cells stably expressing ATeam1.03 in the cytoplasm (cyt-ATeam) and measured FRET signals. Twenty-four hours after treatment with MPP +, a decrease in the FRET ratio was clearly evident. Additions of KUS121, KUS187, or esculetin significantly prevented the decrease of FRET signals. In accord with these changes in FRET signals (Fig. 5g, h), changes in ATP levels were confirmed by classic luciferase activity-based methods (Fig. 5i, Fig. S8). We also created PC12 cells stably expressing ATeam1.03 in the mitochondria (mit-ATeam). Twenty-four hours after treatment with MPP +, a decrease in the FRET ratio was clearly evident in the mitochondria. Additions of KUS121 and KUS187 significantly mitigated the decrease (Fig. S9). By contrast, esculetin further enhanced the decrease (Fig. S9), indicating that KUSs and esculetin oppositely affect mitochondrial ATP levels. These results supported the notion that the maintenance of cytoplasmic ATP levels is more profoundly coupled with the mitigation of ER stress, and thereby with the therapeutic efficacies, than the maintenance of mitochondrial ATP levels.

### 3.3 Effects of KUSs and Esculetin on MPP +-induced Abnormal Features of Mitochondria in PC12 Cells

**Fig. 6:**
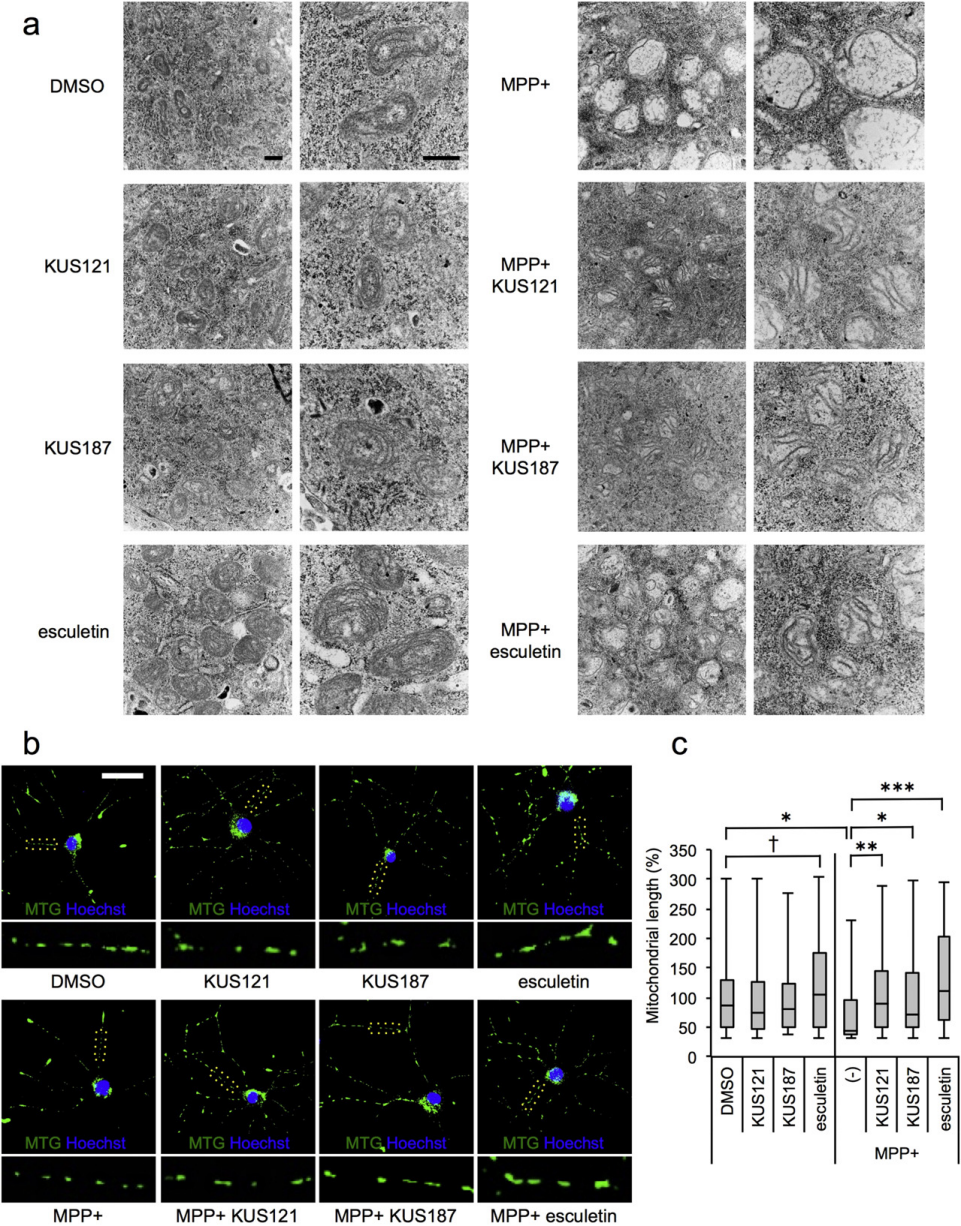
Efficacies of KUSs and esculetin on mitochondrial morphology in the MPP +-induced Parkinson's disease cell culture model. (a) Transmission electron microscopy analyses were performed. Representative images are shown. Neuronally differentiated PC12 cells were incubated for 24 h in the absence (DMSO) or the presence of 50 μM KUSs or 50 μM esculetin, and then further incubated with MPP + for 24 h. Scale bar, 500 nm. (b) Fluorescence microscopy analyses were performed. Representative images of neuronally differentiated PC12 cells are shown. Enlarged portions of neurites (surrounded by yellow dots) are shown below the images. Neuronally differentiated PC12 cells were cultured with the conditions shown in (a). Living cells were incubated with 50 nM MitoTracker Green FM (MTG) and Hoechst33342 (Hoechst) for 30 min, and images were taken by fluorescence microscopy. Scale bar, 50 μm. (c) Quantification of mitochondrial lengths in neurites. Mitochondrial lengths in neurites in (b) were analyzed using MetaMorph (Molecular Devices) and ImageJ software (National Institutes of Health), with mean mitochondrial lengths for DMSO alone set at 100%, and box-and-whisker plots of mitochondrial lengths are shown. The black bars indicate the medians, boxes are 25th–75th quartiles, and whiskers indicate the minimum and maximum values. Images of mitochondria were analyzed for each condition, as follows: DMSO, 80 mitochondria (mt); KUS121, 159 mt; KUS187, 77 mt; esculetin, 86 mt; MPP +, 51 mt; MPP + KUS121, 100 mt; MPP + KUS187, 66 mt; MPP + esculetin, 107 mt. †*P* < 0.05, ANOVA with Games-Howell post-hoc test, vs. DMSO (among non-MPP +-treated groups). *P < 0.05, **P < 0.01, ***P < 0.001, ANOVA with Games-Howell post-hoc test, vs. MPP + alone (-) (among MPP +-treated groups and control (DMSO)).

Given that MPP + is an inhibitor of mitochondrial respiratory chain complex I, and it has been reported that ERR activation leads to mitochondrial biogenesis (Audet-walsh and Giguère, 2015, Dufour et al., 2007, Huss et al., 2004), we thus next examined mitochondrial morphology. Electron microscopic analyses revealed that treatment with esculetin, but not KUS121 or KUS187, appeared to enhance mitochondrial biogenesis, which was substantiated by the presence of mitochondria with high-density cristae, as compared with non-treated cells (Fig. 6a). Consistent with this, neither KUS121 nor KUS187 was capable of transactivating any of the ERRs (Fig. S10). Addition of MPP +, however, induced loss of cristae and vacuolation of mitochondria. These abnormal phenotypes were partly, if not completely, prevented by KUSs or esculetin (Fig. 6a). Immunochemical analyses showed that esculetin, but not KUS121 or KUS187, elongated the lengths of mitochondria in neurites. Addition of MPP + reduced the lengths of the mitochondria, and KUSs or esculetin treatment was able to prevent the reduction (Fig. 6b, c).

We then examined the total mass of mitochondria by measuring intensities of MitoTracker Green FM (Cottet-Rousselle et al., 2011). Consistent with the notion that esculetin promotes mitogenesis (Audet-walsh and Giguère, 2015, Dufour et al., 2007, Huss et al., 2004), esculetin, but not KUS121 or KUS187, increased the total mitochondrial mass, regardless of the presence or absence of MPP + (Fig. S11a). In addition, esculetin, but not KUS121 or KUS187, could mitigate MPP +-induced ROS production, which was consistent with previous reports (Kim et al., 2008, Subramaniam and Ellis, 2013, Subramaniam and Ellis, 2016) (Fig. S11b). By contrast, KUS121 and KUS187, but not esculetin, could prevent the decrease of MPP +-induced mitochondrial membrane potential (Fig. S11c). Thus, with respect to mitochondrial mass, inhibition of ROS production, and maintenance of mitochondrial membrane potential, KUSs and esculetin produced different effects, demonstrating that these effects are not commonly correlated to the efficacies of KUSs and esculetin.

We also examined the protein levels of mitofusin 2 (Mfn2) and dynamin-related protein 1 (Drp1), which play crucial roles in mitochondrial fusion and fission, respectively (Mishra and Chan, 2016, Westermann, 2010). We found that Mfn2 and Drp1 levels remained unchanged regardless of the treatment-even MPP + alone did not alter the respective protein levels (Fig. S12a). Addition of MPP + appeared to induce autophagy or mitophagy, based on the decrease of p62 levels and by the increase of LC3 II. Addition of MPP + appeared to reduce parkin levels and to increase PINK1 levels; both proteins have been reported to be crucial in mitophagy (Nguyen et al., 2016, Pickrell and Youle, 2015, Youle and Narendra, 2011). KUSs treatment prevented all of these changes; esculetin treatment also prevented most of the changes, except the increase of PINK1 levels (Fig. S12b). These results indicate that the effects on mitochondrial dynamics and mitophagy are not likely to be common mechanisms underlying the efficacies of KUSs and esculetin.

### 3.4 KUSs and Esculetin Prevent MPP +-induced Cell Death in Primary Cultures of Dopaminergic Neurons

**Fig. 7:**
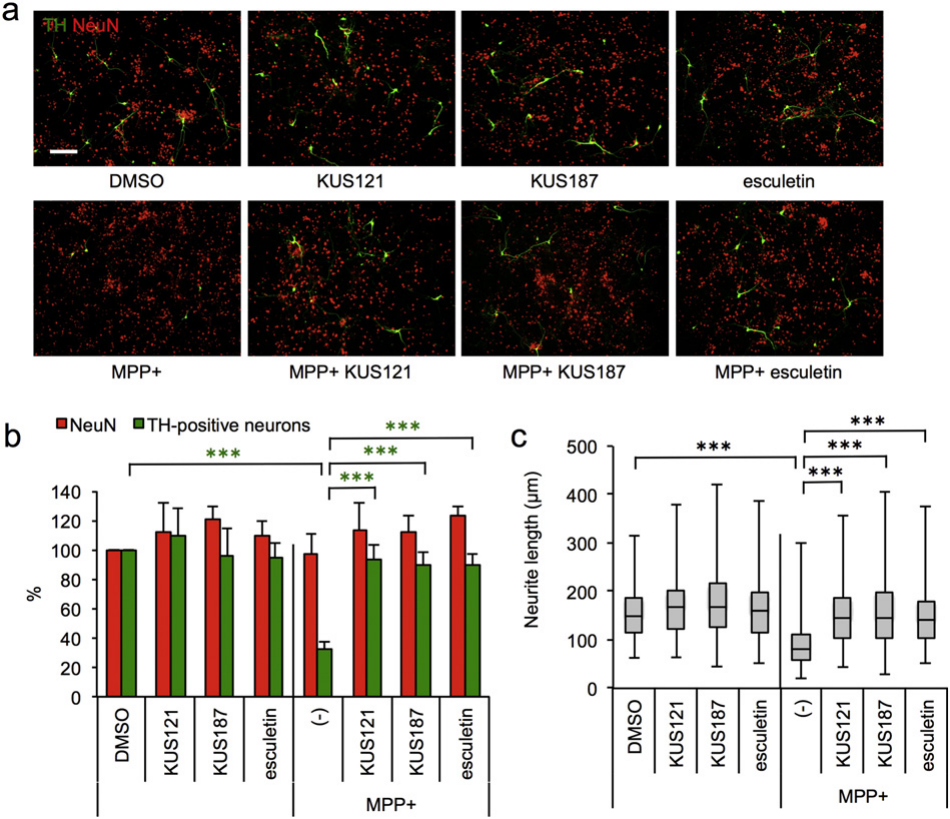
Efficacies of KUSs and esculetin in the MPP +-induced Parkinson's disease model, using primary cultures of dopaminergic neurons. (a) Effects of KUSs and esculetin on the prevention of cell death induced by MPP + in primary mesencephalic cultures. Primary mesencephalic cultures were incubated for 24 h in the absence (DMSO) or the presence of 25 μM KUSs or 25 μM esculetin, and then further incubated with 10 μM MPP + for 48 h. Then, neurons were doubly stained with an anti-tyrosine hydroxylase (TH) antibody (green) for dopaminergic neurons and an anti-NeuN antibody (red) for matured neurons. Representative images captured with a BZ-X700 fluorescence microscope (KEYENCE), are shown. Scale bar, 200 μm. (b) Quantification of TH-positive neurons and NeuN-positive neurons in (a). Mean values of relative neuron numbers are shown, with values for DMSO alone set at 100%. Error bars indicate standard deviations. Statistical significances are shown among MPP +-treated groups and control (DMSO). ***P < 0.001, ANOVA with Games-Howell post-hoc test, vs. MPP + alone (-). (c) Quantification of neurite lengths in TH-positive neurons in images captured with a BZ-X700 fluorescence microscope (KEYENCE). Neurite lengths were analyzed for each condition, as follows: DMSO, 182 neurites; KUS121, 175 neurites; KUS187, 175 neurites; esculetin, 128 neurites; MPP +, 86 neurites; MPP + KUS121, 159 neurites; MPP + KUS187, 142 neurites; MPP + esculetin, 125 neurites. Box-and-whisker plots of neurite lengths are shown. The black bars indicate the medians, boxes are 25th–75th quartiles, and whiskers indicate the minimum and maximum values. Statistical significances are shown among MPP +-treated groups and control (DMSO). ***P < 0.001, ANOVA with Games-Howell post-hoc test, vs. MPP + alone (-).

PC12 was originally established from a pheochromocytoma of adrenal origin, and neuronally differentiated PC12 cells are estimated to have characteristics of sympathetic neurons (Greene and Tischler, 1976), and possess some characteristics of dopaminergic neurons, as evidenced by their expression of tyrosine hydroxylase and their ability to incorporate MPP +, etc. (Grima et al., 1985, Jin et al., 2007, Kumer and Vrana, 1996). In order to further confirm the neuroprotective efficacies of KUSs and esculetin on dopaminergic neurons, we examined their effects on primary cultures of mesencephalic neurons with or without MPP + treatment. KUS121, KUS187, or esculetin treatment alone did not affect the survival of NeuN (Fox-3)- or tyrosine hydroxylase-positive neurons (Fig. 7a, b). Without KUSs or esculetin, MPP + specifically killed dopaminergic neurons, as seen by the specific decrease of tyrosine hydroxylase-positive neurons (Fig. 7a, b). It is notable that MPP + apparently reduced neurite lengths of remaining dopaminergic neurons (Fig. 7c). Addition of KUS121, KUS187, or esculetin significantly protected the dopaminergic neurons from the MPP +-induced cell death and from the MPP +-induced neurite shrinkage (Fig. 7).

### 3.5 Effects of KUS121 and Esculetin on Mouse Models of Parkinson's Disease

In order to evaluate the potential efficacies of KUS121 and esculetin on Parkinson's disease, we first set up a reproducible protocol for MPTP-induced Parkinson's disease in mice. We tested several different protocols and ultimately found that the following protocol could reproducibly create Parkinson's disease phenotypes in mice: 20 mg/kg MPTP together with 250 mg/kg probenecid (CAS No. 57-66-9) were injected intraperitoneally twice per week for 5 months. Probenecid has the ability to inhibit renal excretion of several chemicals, including MPTP, and thereby raise the blood concentrations of such chemicals (Lau et al., 1990, Masereeuw et al., 2000). Hereinafter, we refer to these mice as MPTP-treated mice (MPTP mice).

**Fig. 8:**
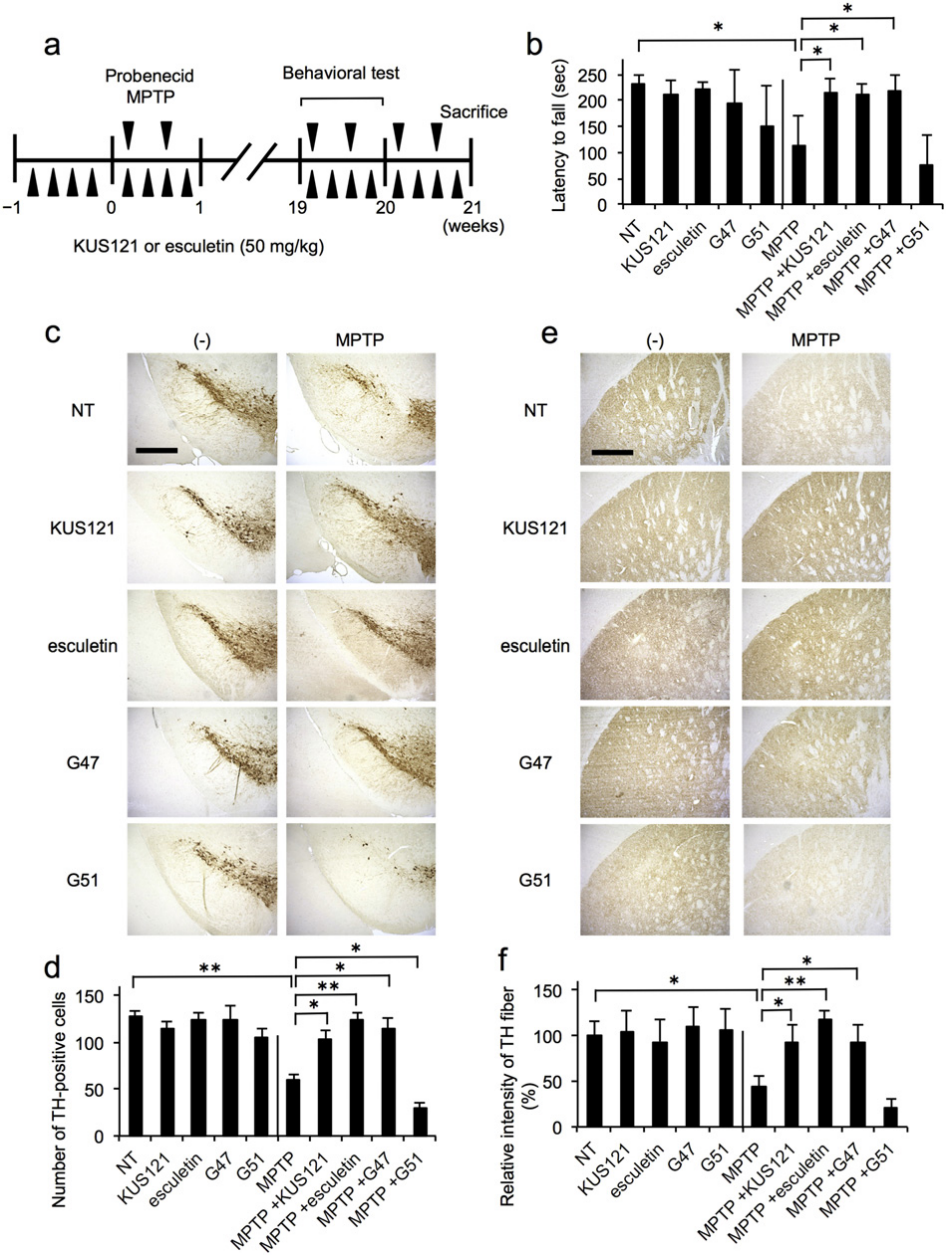
Efficacies of KUS121 and esculetin on MPTP-induced Parkinson's disease model mice. (a) Schematic drawing of experimental schedules for creation and treatment of chronic MPTP-induced Parkinson's disease model mice. See details in “Materials and Methods”. (b) The results of the rotarod test performed at 20 weeks. Mean times (s) to fall with standard deviations are shown. No drug-treated control (NT; *n* = 7), KUS121-treated (KUS121; *n* = 6), esculetin-treated (esculetin; *n* = 6), GSK4716-treated (G47; *n* = 5), GSK5182-treated (G51; *n* = 5). MPTP-treated (MPTP; *n* = 7), MPTP + KUS121-treated (MPTP + KUS121; *n* = 7), MPTP + esculetin-treated (MPTP + esculetin; *n* = 7), MPTP + GSK4716-treated (MPTP + G47; *n* = 7), and MPTP + GSK5182-treated (MPTP + G51; *n* = 7). Error bars indicate standard deviations. Statistical significances are shown among MPTP-treated and no drug-treated control (NT) groups. *P < 0.05, ANOVA with Games-Howell post-hoc test, vs. MPTP alone (MPTP). The results of the rotarod test performed at 0, 4, 8, 12, and 16 weeks are shown in Fig. S19. (c) Immunohistochemical analyses of coronal sections of the mouse substantia nigra, stained with an anti-tyrosine hydroxylase (TH) antibody, followed by the ABC method. Representative images are shown. Scale bar, 400 μm. (d) Quantification of TH-positive neurons in (c). Error bars indicate standard deviations. Statistical significances are shown among MPTP-treated and no drug-treated control (NT) groups. *P < 0.05, **P < 0.01, ANOVA with Games-Howell post-hoc test (*n* = 3), vs. MPTP alone (MPTP). (e) Immunohistochemical analyses of coronal sections of the mouse striatum, stained by an anti-TH antibody, followed by the ABC method. Representative images are shown. Scale bar, 400 μm. (f) Quantification of TH-positive signals in (e). Relative intensities of the TH-positive signals are shown, with values for no drug-treated control (NT) group set at 100%. Error bars indicate standard deviations. Statistical significances are shown among MPTP-treated and no drug-treated control (NT) groups. *P < 0.05, **P < 0.01, ANOVA with Games-Howell post-hoc test (*n* = 4), vs. MPTP alone (MPTP).

We then evaluated the efficacies of KUS121 and esculetin on MPTP mice. For the evaluation, we administrated vehicle or 50 mg/kg of KUS121 or esculetin, 4 times per week for 5 months concurrent with MPTP treatment (Fig. 8a). As controls, we also administrated GSK4716 and GSK5182, 4 times per week for the 5 months. As a result, MPTP treatments did not induce any difference in the grip strength test (Fig. S13a), wire hang test (Fig. S13b), treadmill test (Fig. S13c), or open field test (Fig. S13d–h), as compared with no drug-treated control (NT) mice. We observed neither improvement nor exacerbation by KUS121, esculetin, GSK4716, or GSK5182 with these tests in MPTP mice. Importantly, however, MPTP mice stayed on rotor rods for significantly shorter durations, as compared with NT mice, and administration of KUS121, esculetin, or GSK4716 allowed the MPTP mice to stay on rotor rods for durations that were comparable to those of the NT mice (Fig. 8b).

These results implied that KUS121, esculetin, and GSK4716 had neuroprotective effects on dopaminergic neurons of the substantia nigra in MPTP mice. Histochemical examinations of midbrains from these mice revealed that MPTP treatments significantly reduced the numbers of tyrosine hydroxylase-positive neurons, namely dopaminergic neurons, in the midbrains, especially the substantia nigra pars compacta. Administrations of KUS121, esculetin, or GSK4716 significantly mitigated the loss of the dopaminergic neurons (Fig. 8c, d). Consistent with these results, immunohistochemical analyses of the striatum showed a reduction of tyrosine hydroxylase levels in MPTP mice, and the reduction was prevented by the administration of KUS121, esculetin, or GSK4716, but not GSK5182 (Fig. 8e, f). These results were further confirmed by western blot analyses of midbrain extracts from these mice (Fig. S14).

Very similar results were obtained from the analyses of rotenone-induced Parkinson's disease mice (Dauer and Przedborski, 2003, Inden et al., 2011) (Fig. S15). One clear difference between the two models was the following: rotenone-induced Parkinson's disease mice, but not MPTP-induced Parkinson's disease mice, manifested less movement in the open field test (Figs. S13e, S15f). Importantly, the deficit was prevented by the addition of KUS121 or esculetin (Fig. S15f).

### 3.6 KUS121 and Esculetin Mitigate MPTP-induced α-Synuclein Accumulation, ATP Depletion, and ER Stress

**Fig. 9:**
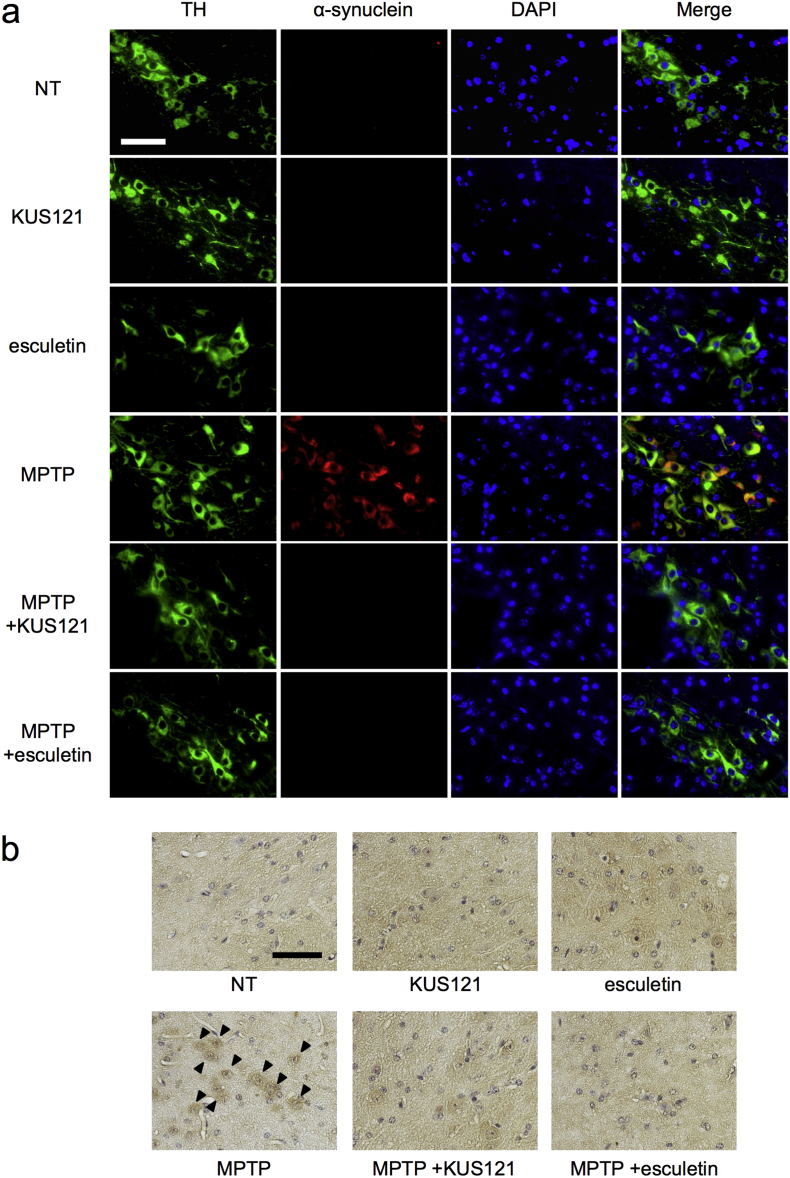
Efficacies of KUS121 and esculetin on α-synuclein levels in the substantia nigra of MPTP-induced Parkinson's disease model mice. (a) Triple labeling of dopaminergic neurons of the mouse substantia nigra with an anti-tyrosine hydroxylase (TH) antibody (green), an anti-α-synuclein antibody (red), and DAPI (blue). Representative images are shown. Scale bar, 50 μm. (b) Immunohistochemical analyses of coronal sections of the mouse substantia nigra, stained with an anti-α-synuclein antibody, followed by the ABC method. Nuclei were counter-stained with hematoxylin. α-Synuclein-positive cells are indicated by black arrowheads. Mouse brains were dissected at 5 months after the start of MPTP treatment. Scale bar, 50 μm. (For interpretation of the references to colour in this figure legend, the reader is referred to the web version of this article.)

Next, we examined the link between α-synuclein and ATP decrease. In the dopaminergic neurons of the substantia nigra of no drug-treated control (NT) mice and KUS121- or esculetin-treated mice, we were not able to detect the expression of α-synuclein proteins. However, MPTP treatment dramatically provoked elevated α-synuclein levels, and KUS121 or esculetin treatment almost completely abrogated the accumulation of α-synuclein protein (Fig. 9).

**Fig. 10:**
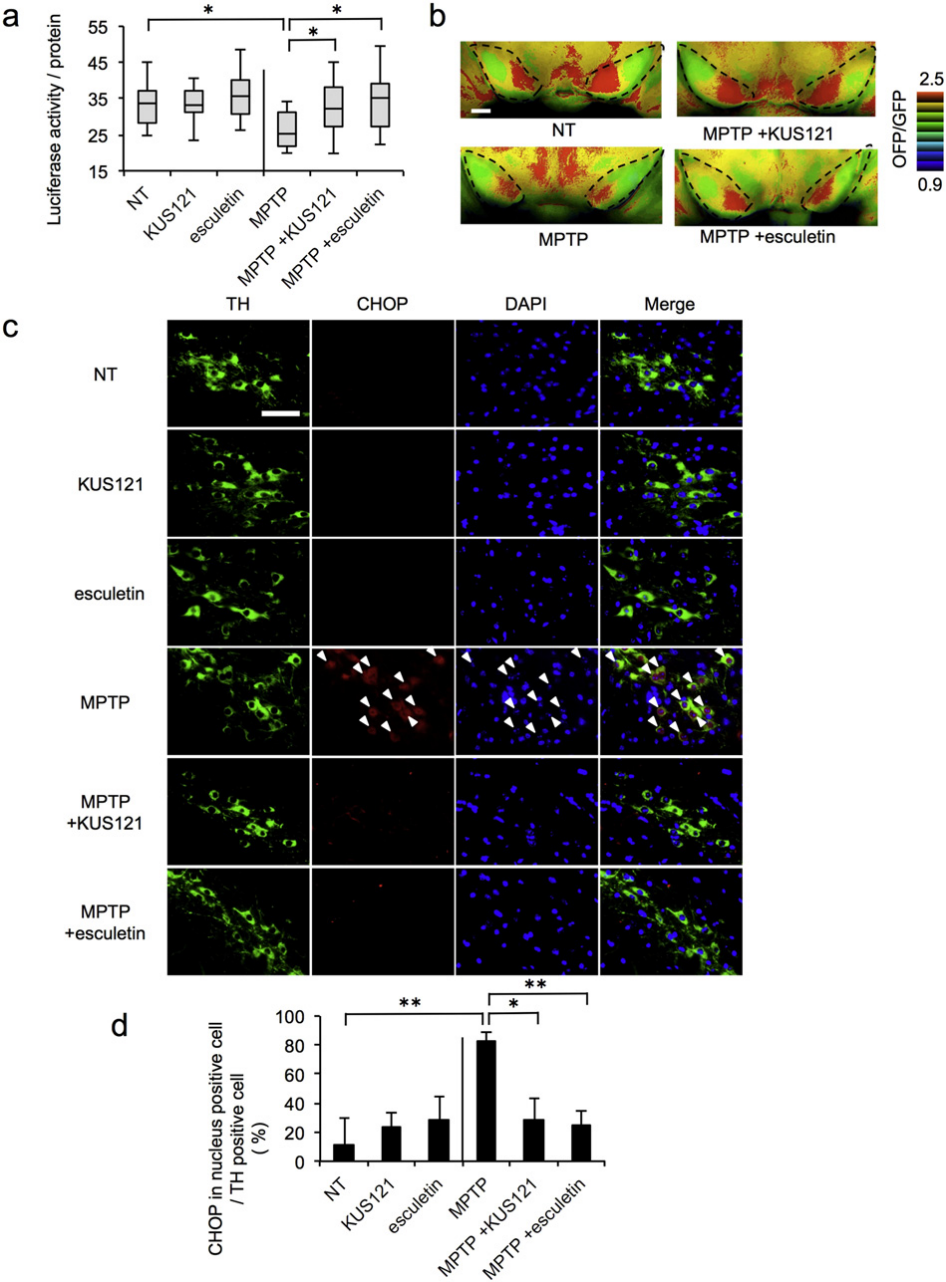
Efficacies of KUS121 and esculetin on ER stress and ATP depletion in the substantia nigra of MPTP-induced Parkinson's disease model mice. (a) Quantitation of ATP levels in mouse midbrains containing the substantia nigra and striatum. No drug-treated control mice (NT; *n* = 15), KUS121-treated (*n* = 14), esculetin-treated (*n* = 14), MPTP-treated (MPTP; *n* = 15), MPTP + KUS121-treated (MPTP + KUS121; *n* = 15), and MPTP + esculetin-treated (MPTP + esculetin; *n* = 15) mice were dissected one month after the start of MPTP treatment. ATP levels and protein amounts were determined, and values (luciferase activities) of ATP levels, normalized by protein levels, were calculated and are shown as box-and-whisker plots. The black bars indicate the medians, boxes are 25th–75th quartiles, and whiskers indicate the minimum and maximum values. Statistical significances are shown among MPTP-treated and no drug-treated control (NT) groups. *P < 0.05, ANOVA with Games-Howell post-hoc test, vs. MPTP alone (MPTP). (b) Representative ratiometric pseudocolor FRET images of coronal slice sections of midbrains of Go-ATeam2 mice. Mouse brains were dissected from non-treated Go-ATeam2 control mice (NT), and Go-ATeam2 mice treated with MPTP alone (MPTP), with MPTP + KUS121 (MPTP + KUS121), or with MPTP + esculetin (MPTP + esculetin), at 1 month after starting MPTP treatment. OFP/GFP emission ratios of Go-ATeam2 in the slices of midbrains were then calculated from captured images. Representative images are shown. Areas marked by black dots indicate the substantia nigra. Scale bar, 400 μm. (c) Triple labeling of dopaminergic neurons of the mouse substantia nigra with an anti-tyrosine hydroxylase (TH) antibody (green), an anti-CHOP antibody (red), and DAPI (blue). Mouse brains were dissected from non-treated control mice without additional treatment (NT), or mice treated with KUS121 (KUS121) or esculetin (esculetin), and MPTP mice without additional treatment (MPTP) or treated with KUS121 (MPTP + KUS121) or esculetin (MPTP + esculetin), at 5 months after the start of MPTP treatment. CHOP expression increased in the dopaminergic neurons in the substantia nigra pars compacta of MPTP-treated mice (white arrowheads). Representative images are shown. Scale bar, 50 μm. (d) Quantitation of the percentage of TH-positive cells with CHOP-positive nuclei in (c). Error bars indicate standard deviations. Statistical significances are shown among MPTP-treated and no drug-treated control (NT) groups. *P < 0.05, **P < 0.01, ANOVA with Games-Howell post-hoc test (*n* = 3), vs. MPTP alone (MPTP). (For interpretation of the references to colour in this figure legend, the reader is referred to the web version of this article.)

We then measured ATP levels in brain sections of control and MPTP mice. Consistent with the results from PC12 cells and the mouse phenotypes mentioned above, ATP levels in the midbrain extracts were significantly decreased in MPTP mice, as compared with NT mice, and the reduction was prevented by the administration of KUS121 and esculetin (Fig. 10a). These results were confirmed by the FRET signals in midbrain slices of Go-ATeam2 mice (Fig. 10b).

Finally, we analyzed ER stress levels in these mice by immunohistochemical analyses using an anti-CHOP antibody. In NT mice, approximately 10% of dopaminergic neurons (tyrosine hydroxylase-positive cells) in the substantia nigra showed CHOP expression. As expected, in the substantia nigra from MPTP mice, approximately 80% or more of the extant dopaminergic neurons were CHOP-positive, and the administration of KUS121 and esculetin reduced the relative abundance of CHOP-positive cells among dopaminergic neurons to approximately 30% or less (Fig. 10c, d, Fig. S16).

## 4 Discussion

In many incurable human diseases, including neurodegenerative diseases (e.g. Alzheimer's disease, Parkinson's disease, Amyotrophic lateral sclerosis (ALS), polyglutamine diseases, etc.), the most common pathology is early cell death in the affected organs, or specific brain areas in the case of neurodegenerative diseases. ATP levels would be one of the most important parameters representing overall health conditions of the individual cell, and an ATP decrease appears as the most common phenotype of dying cells. Nutrients are major sources of ATP, and among them glucose is known to be the most potent ATP-producing nutrient. However, high levels of glucose in serum, by themselves, do not raise or even maintain ATP levels in cells, especially in certain pathological conditions, e.g. in diabetes mellitus and in the Parkinson's disease model shown in this study. It is notable that diabetes mellitus is a risk factor for Alzheimer's disease, indicating that high serum glucose levels do not prevent or might even promote neuronal cell death in Alzheimer's disease (Cheng et al., 2012, Stewart and Liolitsa, 1999).

We have long been searching for chemicals that could prevent such cell death in vivo. For this purpose, we have hypothesized that such chemicals would maintain cellular ATP levels under these pathological conditions. In order to address this hypothesis, we developed KUSs as specific inhibitors of the ATPase activity of VCP, the most abundant soluble ATPase in essentially all types of cells. Indeed, KUSs could prevent ATP decreases provoked by several cell death-inducing insults, e.g. glucose or serum deprivation, mitochondrial respiratory chain inhibition, etc., leading to the suppression of ER stress and cell death in cultured cells (Ikeda et al., 2014, Nakano et al., 2016). Furthermore, KUS administration was able to inhibit in vivo retinal neuronal cell death and mitigate disease phenotypes in several mouse models of retinal diseases, e.g. retinitis pigmentosa, glaucoma, and ischemic retinal disease (Hasegawa et al., 2016b, Hata et al., 2017, Ikeda et al., 2014, Nakano et al., 2016). On the other hand, our previous results indicated that activation of nuclear receptors, specifically ERRs, would lead to increased ATP production in the targeted cells or organs (Kamei et al., 2003). We, thus, searched for synthetic agonistic ligands of ERRs and showed in this study that esculetin could function as an ERR agonist. To the best of our knowledge, this is the first small chemical that functions as an ERR agonist for all three ERRs. As expected, esculetin treatment dose-dependently induced an increase in glucose and oxygen consumption in neuronally differentiated PC12 cells, eventually leading to high ATP levels, up to 180% relative to the non-treated cells (Fig. 2b, c, e, Fig. S1). We thus call these chemicals, as a whole, “ATP regulators”.

Pathological hallmarks of Parkinson's disease are the loss of dopaminergic neurons in the substantia nigra and the presence of Lewy bodies, protein aggregates of α-synuclein, in the extant dopaminergic neurons. To date, 18 genetic loci have been linked to familial human Parkinson's disease, and are named PARK1 to PARK18 (Klein and Westenberger, 2012, Lin and Farrer, 2014). PARK1 is the *α-synuclein* gene itself. These causative genes have been overexpressed, in the case of dominant inheritance, or knocked out, in the case of recessive inheritance, to produce mouse models for Parkinson's disease. However, no such mice have yet successfully manifested either the loss of dopaminergic neurons or Parkinson's disease-like phenotypes. On the other hand, there are established chemically induced Parkinson's disease rodent models, using MPTP, rotenone, 6-hydroxydopamine, etc. (Dauer and Przedborski, 2003, Lindholm et al., 2016). As contrasted with genetically manipulated models, these models did manifest prominent loss of dopaminergic neurons. Since we are interested in the death of dopaminergic neurons and their protection, we used chemically induced Parkinson's disease models in this study.

Beyond our expectations, it was evident that KUSs and esculetin behave very similarly in preventing ATP decrease, ER stress, and cell death, in cell culture as well as in a mouse model of Parkinson's disease brought on by administration of MPTP. In neuronally differentiated PC12 cells and in primary cultures of dopaminergic neurons, MPP + treatment led to decreased ATP levels, induction of CHOP expression (ER stress), and eventually cell death; yet both KUSs and esculetin suppressed all of these (Fig. 3, Fig. 4, Fig. 5g–i, 7, Figs. S2–S5). Moreover, administration of KUS121 or esculetin concurrently with MPTP maintained ATP levels and prevented ER stress in dopaminergic neurons in the substantia nigra, and ultimately suppressed the manifestation of Parkinson's disease phenotypes, as seen with the rotarod test (Fig. 8, Fig. 10). We confirmed very similar efficacies of KUS121 and esculetin in protecting dopaminergic neurons in the substantia nigra of a rotenone-induced mouse Parkinson's disease model (Fig. S15). We also observed that administration of GSK4716, an ERRγ-specific agonist, could mitigate the Parkinson's disease phenotypes. On the other hand, with an ERRγ-specific inverse-agonist, GSK5182, Parkinson's disease features were either unchanged or exacerbated (Fig. 8).

The most surprising data for us was the behavior of α-synuclein protein; it accumulated in the dopaminergic neurons of the substantia nigra in response to MPTP treatment, and it failed to accumulate during the treatment with KUSs and esculetin (Fig. 9). Related with this, very recently it has been reported that ATP itself has chaperone activities (Patel et al., 2017); ATP may help to prevent misfolding or accumulation of α-synuclein. It has also been reported that α-synuclein acts on mitochondria and stimulates ATP synthesis (Ludtmann et al., 2016). These lines of evidence emphasize a close link between α-synuclein protein levels and ATP levels. It is tempting to speculate that Lewy bodies might be a marker of a prior drop in ATP levels.

In addition to the activities shown in this study, esculetin has recently been shown to have cell-protective effects, and to mitigate MPTP-induced Parkinson's disease phenotypes in mice (Subramaniam and Ellis, 2013). These effects were proposed to be mediated by the reduction of oxidative stress caused by reactive oxygen species or nitrosylation (Kim et al., 2008, Subramaniam and Ellis, 2013, Subramaniam and Ellis, 2016). These results further support a therapeutic potential of esculetin for the treatment of Parkinson's disease. Esculetin has also been shown to possess cell death-inducing activities in several cultured cancerous cells (Arora et al., 2016, Jeon et al., 2015, Wang et al., 2015), and thus long-term esculetin treatment, if approved, may have the added benefit of reducing cancer occurrence.

We have shown that KUSs can protect retinal neurons from death in mouse models of retinitis pigmentosa, glaucoma, and ischemic retinal disease, and we also have repeatedly discussed mechanisms that underlie the profound links between ATP decrease, ER stress, and cell death in these eye diseases (Hasegawa et al., 2016b, Hata et al., 2017, Ikeda et al., 2014, Nakano et al., 2016). In this study, we further showed that KUSs and esculetin can protect dopaminergic neurons from MPTP- or rotenone-induced toxicity in the respective mouse models of Parkinson's disease (Fig. 8, Fig. 10, Figs. S14, S15, S19). From these results, we propose a model for the pathological features of these disorders, in which ATP decrease or depletion is a common central phenomenon, even though each disease has a unique etiology. An ATP decrease in early stages would diminish the functions of affected cells or organs, and then in later stages it would result in cell death or organ failure, culminating in overt disease phenotypes. Furthermore, this model suggests potential therapeutic strategies commonly applicable to these disorders. Namely, maintenance of ATP levels, by either inhibiting ATP consumption or enhancing ATP production, or both, would slow or stop the disease progression. Functional recovery of the remaining cells might even ameliorate the disease phenotypes.

In conclusion, ATP maintenance by “ATP regulators”, i.e., KUSs and esculetin (or ERR agonists), showed significant efficacies for neuroprotection in the MPTP- and rotenone-induced Parkinson's disease model mice. Given that many incurable human disorders, e.g. neurodegenerative diseases, ischemic diseases, etc., also manifest early cell death in the affected organs, ATP maintenance by ATP regulators could also provide a strategy for cell protection in these disorders.

## Funding Sources

This research was supported in part by research grants from the Mitsubishi Foundation (28107), the Ministry of Education, Culture, Sports, Science and Technology, Japan (16H05151), and by Solution-Oriented Research for Science and Technology (SORST-H16-3) from the Japan Science and Technology Agency (JST). And this research was supported by the Platform Project for Supporting Drug Discovery and Life Science Research (Platform for Dynamic Approaches to Living Systems) from the Japan Agency for Medical Research and Development (AMED) (16am0101009j0005).

## Appendix A Supplementary data

Supplementary Materials. Image 1

## References

[bb0005] Alaynick W.A., Kondo R.P., Xie W., He W., Dufour C.R., Downes M., Jonker J.W., Giles W., Naviaux R.K., Giguère V. (2007). ERRγ directs and maintains the transition to oxidative metabolism in the postnatal heart. Cell Metab..

[bb0010] Arora R., Sawney S., Saini V., Steffi C., Tiwari M., Saluja D. (2016). Esculetin induces antiproliferative and apoptotic response in pancreatic cancer cells by directly binding to KEAP1. Mol. Cancer.

[bb0015] Audet-walsh É., Giguère V. (2015). The multiple universes of estrogen-related receptor α and γ in metabolic control and related diseases. Acta Pharmacol. Sin..

[bb0020] Cheng G., Huang C., Deng H., Wang H. (2012). Diabetes as a risk factor for dementia and mild cognitive impairment: a meta-analysis of longitudinal studies. Intern. Med. J..

[bb0025] Cottet-Rousselle C., Ronot X., Leverve X., Mayol J.F. (2011). Cytometric assessment of mitochondria using fluorescent probes. Cytometry A.

[bb0030] Dauer W., Przedborski S. (2003). Parkinson's disease: mechanisms and models. Neuron.

[bb0035] Davis G.C., Williams A.C., Markey S.P., Ebert M.H., Caine E.D., Reichert C.M., Kopin I.J. (1979). Chronic Parkinsonism secondary to intravenous injection of meperidine analogues. Psychiatry Res..

[bb0040] Dettmer U., Newman A.J., von Saucken V.E., Bartels T., Selkoe D. (2015). KTKEGV repeat motifs are key mediators of normal α-synuclein tetramerization: their mutation causes excess monomers and neurotoxicity. Proc. Natl. Acad. Sci..

[bb0045] Dufour C.R., Wilson B.J., Huss J.M., Kelly D.P., Alaynick W.A., Downes M., Evans R.M., Blanchette M., Giguère V. (2007). Genome-wide orchestration of cardiac functions by the orphan nuclear receptors ERRα and γ. Cell Metab..

[bb0050] Gaven F., Marin P., Claeysen S. (2014). Primary culture of mouse dopaminergic neurons. J. Vis. Exp..

[bb0055] Greene L.A., Tischler A.S. (1976). Establishment of a noradrenergic clonal line of rat adrenal pheochromocytoma cells which respond to nerve growth factor. Proc. Natl. Acad. Sci..

[bb0060] Grima B., Lamouroux A., Blanot F., Biguet N.F., Mallet J. (1985). Complete coding sequence of rat tyrosine hydroxylase mRNA. Proc. Natl. Acad. Sci..

[bb0065] Halliday G.M., Leverenz J.B., Schneider J.S., Adler C.H. (2014). The neurobiological basis of cognitive impairment in Parkinson's disease. Mov. Disord..

[bb0070] Hasegawa K., Yasuda T., Shiraishi C., Fujiwara K., Przedborski S., Mochizuki H., Yoshikawa K. (2016). Promotion of mitochondrial biogenesis by necdin protects neurons against mitochondrial insults. Nat. Commun..

[bb0075] Hasegawa T., Muraoka Y., Ikeda H.O., Tsuruyama T., Kondo M., Terasaki H., Kakizuka A., Yoshimura N. (2016). Neuoroprotective efficacies by KUS121, a VCP modulator, on animal models of retinal degeneration. Sci Rep.

[bb0080] Hata M., Ikeda H.O., Kikkawa C., Iwai S., Muraoka Y., Hasegawa T., Kakizuka A., Yoshimura N. (2017). KUS121, a VCP modulator, attenuates ischemic retinal cell death via suppressing endoplasmic reticulum stress. Sci Rep.

[bb0085] Higashiyama H., Hirose F., Yamaguchi M., Inoue Y.H., Fujikake N., Matsukage A., Kakizuka A. (2002). Identification of ter94, Drosophila VCP, as a modulator of polyglutamine-induced neurodegeneration. Cell Death Differ..

[bb0090] Huss J.M., Torra I.P., Staels B., Giguère V., Kelly D.P. (2004). Estrogen-related receptor α directs peroxisome proliferator-activated receptor α signaling in the transcriptional control of energy metabolism in cardiac and skeletal muscle. Mol. Cell. Biol..

[bb0095] Ikeda H., Yamaguchi M., Sugai S., Aze Y., Narumiya S., Kakizuka A. (1996). Expanded polyglutamine in the Machado-Joseph disease protein induces cell death in vitro and in vivo. Nat. Genet..

[bb0100] Ikeda H.O., Sasaoka N., Koike M., Nakano N., Muraoka Y., Toda Y., Fuchigami T., Shudo T., Iwata A., Hori S. (2014). Novel VCP modulators mitigate major pathologies of rd10, a mouse model of retinitis pigmentosa. Sci Rep.

[bb0105] Imamura H., Nhat K.P., Togawa H., Saito K., Iino R., Kato-Yamada Y., Nagai T., Noji H. (2009). Visualization of ATP levels inside single living cells with fluorescence resonance energy transfer-based genetically encoded indicators. Proc. Natl. Acad. Sci..

[bb0110] Inden M., Kitamura Y., Abe M., Tamaki A., Takata K., Taniguchi T. (2011). Parkinsonian rotenone mouse model: reevaluation of long-term administration of rotenone in C57BL/6 mice. Biol. Pharm. Bull..

[bb0115] Iwakura Y., Zheng Y., Sibilia M., Abe Y., Piao Y.S., Yokomaku D., Wang R., Ishizuka Y., Takei N., Nawa H. (2011). Qualitative and quantitative re-evaluation of epidermal growth factor-ErbB1 action on developing midbrain dopaminergic neurons in vivo and in vitro: target-derived neurotrophic signaling (part 1). J. Neurochem..

[bb0120] Jeon Y.J., Jang J.Y., Shim J.H., Myung P.K., Chae J.I. (2015). Esculetin, a Coumarin derivative, exhibits anti-proliferative and pro-apoptotic activity in G361 human malignant melanoma. J. Cancer Prev..

[bb0125] Jin G.Z., Yin X.J., Yu X.F., Cho S.J., Lee H.S., Lee H.J., Kong I.K. (2007). Enhanced tyrosine hydroxylase expression in PC12 cells co-cultured with feline mesenchymal stem cells. J. Vet. Sci..

[bb0130] Johnson J.O., Mandrioli J., Benatar M., Abramzon Y., Deerlin V.M.V., Trojanowski J.Q., Gibbs J.R., Brunetti M., Gronka S., Wuu J. (2010). Exome sequencing reveals VCP mutations as a cause of familial ALS. Neuron.

[bb0135] Kakizuka A. (1998). Protein precipitation: a common etiology in neurodegenerative disorders?. Trends Genet..

[bb0140] Kamei Y., Ohizumi H., Fujitani Y., Nemoto T., Tanaka T., Takahashi N., Kawada T., Miyoshi M., Ezaki O., Kakizuka A. (2003). PPARγ coactivator 1β/ERR ligand 1 is an ERR protein ligand, whose expression induces a high-energy expenditure and antagonizes obesity. Proc. Natl. Acad. Sci..

[bb0145] Kawaguchi Y., Okamoto T., Taniwaki M., Aizawa M., Inoue M., Katayama S., Kawakami H., Nakamura S., Nishimura M., Akiguchi I. (1994). CAG expansions in a novel gene for Machado-Joseph disease at chromosome 14q32.1. Nat. Genet..

[bb0150] Kim S.H., Kang K.A., Zhang R., Piao M.J., Ko D.O., Wang Z.H., Chae S.W., Kang S.S., Lee K.H., Kang H.K. (2008). Protective effect of esculetin against oxidative stress-induced cell damage via scavenging reactive oxygen species. Acta Pharmacol. Sin..

[bb0155] Kitada T., Asakawa S., Hattori N., Matsumine H., Yamamura Y., Minoshima S., Yokochi M., Mizuno Y., Shimizu N. (1998). Mutations in the parkin gene cause autosomal recessive juvenile parkinsonism. Nature.

[bb0160] Klein C., Westenberger A. (2012). Genetics of Parkinson's disease. Cold Spring Harb. Perspect. Med..

[bb0165] Kumer S.C., Vrana K.E. (1996). Intricate regulation of tyrosine hydroxylase activity and gene expression. J. Neurochem..

[bb0170] Langston J.W., Ballard P., Tetrud J.W., Irwin I. (1983). Chronic Parkinsonism in humans due to a product of meperidine-analog synthesis. Science.

[bb0175] Lau Y.S., Trobough K.L., Crampton J.M., Wilson J.A. (1990). Effects of probenecid on striatal dopamine depletion in acute and long-term 1-methyl-4-phenyl-1, 2, 3, 6-tetrahydropyridine (MPTP)-treated mice. Gen. Pharmacol..

[bb0180] Lin M.K., Farrer M.J. (2014). Genetics and genomics of Parkinson's disease. Genome Med..

[bb0185] Lindholm D., Mäkelä J., Liberto V.D., Mudò G., Belluardo N., Eriksson O., Saarma M. (2016). Parkinson's disease: towards better preclinical models and personalized treatments. Cell. Mol. Life Sci..

[bb0190] Ludtmann M.H., Angelova P.R., Ninkina N.N., Gandhi S., Buchman V.L., Abramov A.Y. (2016). Monomeric alpha-synuclein exerts a physiological role on brain ATP synthase. J. Neurosci..

[bb0195] Manno A., Noguchi M., Fukushi J., Motohashi Y., Kakizuka A. (2010). Enhanced ATPase activities as a primary defect of mutant valosin-containing proteins that cause inclusion body myopathy associated with Paget disease of bone and frontotemporal dementia. Genes Cells.

[bb0200] Masereeuw R., Van Pelt A.P., Van Os S.H., Willems P.H., Smits P., Russel F.G. (2000). Probenecid interferes with renal oxidative metabolism: a potential pitfall in its use as an inhibitor of drug transport. Br. J. Pharmacol..

[bb0205] McCann H., Stevens C.H., Cartwright H., Halliday G.M. (2014). α-Synucleinopathy phenotypes. Parkinsonism Relat. Disord..

[bb0210] Mishra P., Chan D.C. (2016). Metabolic regulation of mitochondrial dynamics. J. Cell Biol..

[bb0215] Mudò G., Mäkelä J., Liberto V.D., Tselykh T.V., Olivieri M., Piepponen P., Eriksson O., Mälkiä A., Bonomo A., Kairisalo M. (2012). Transgenic expression and activation of PGC-1α protect dopaminergic neurons in the MPTP mouse model of Parkinson's disease. Cell. Mol. Life Sci..

[bb0220] Nakano M., Imamura H., Nagai T., Noji H. (2011). Ca^2 +^ regulation of mitochondrial ATP synthesis visualized at the single cell level. ACS Chem. Biol..

[bb0225] Nakano N., Ikeda H.O., Hasegawa T., Muraoka Y., Iwai S., Tsuruyama T., Nakano M., Fuchigami T., Shudo T., Kakizuka A. (2016). Neuroprotective effects of VCP modulators in mouse models of glaucoma. Heliyon.

[bb0230] Nguyen T.N., Padman B.S., Lazarou M. (2016). Deciphering the molecular signals of PINK1/Parkin mitophagy. Trends Cell Biol..

[bb0235] Patel A., Malinovska L., Saha S., Wang J., Alberti S., Krishnan Y., Hyman A.A. (2017). ATP as a biological hydrotrope. Science.

[bb0240] Pickrell A.M., Youle R.J. (2015). The roles of PINK1, parkin, and mitochondrial fidelity in Parkinson's disease. Neuron.

[bb0245] Polymeropoulos M.H., Lavedan C., Leroy E., Ide S.E., Dehejia A., Dutra A., Pike B., Root H., Rubenstein J., Boyer R. (1997). Mutation in the α-synuclein gene identified in families with Parkinson's disease. Science.

[bb0250] Ramsay R.R., Krueger M.J., Youngster S.K., Gluck M.R., Casida J.E., Singer T.P. (1991). Interaction of 1-methyl-4-phenylpyridinium ion (MPP +) and its analogs with the rotenone/piericidin binding site of NADH dehydrogenase. J. Neurochem..

[bb0255] Saitou M., Narumiya S., Kakizuka A. (1994). Alteration of a single amino acid residue in retinoic acid receptor causes dominant-negative phenotype. J. Biol. Chem..

[bb0260] Sasaoka N., Sakamoto M., Kanemori S., Kan M., Tsukano C., Takemoto Y., Kakizuka A. (2014). Long-term oral administration of hop flower extracts mitigates Alzheimer phenotypes in mice. PLoS One.

[bb0265] Sharma M., Ioannidis J.P., Aasly J.O., Annesi G., Brice A., Bertram L., Bozi M., Barcikowska M., Crosiers D., Clarke C.E. (2012). A multi-centre clinico-genetic analysis of VPS35 gene in Parkinson disease indicates reduced penetrance for disease-associated variants. J. Med. Genet..

[bb0270] Sharpe M.A., Livingston A.D., Gist T.L., Ghosh P., Han J., Baskin D.S. (2015). Successful treatment of intracranial glioblastoma xenografts with a monoamine oxidase B-activated pro-drug. EBioMedicine.

[bb0275] Spillantini M.G., Schmidt M.L., Lee V.M.Y., Trojanowski J.Q., Jakes R., Goedert M. (1997). α-Synuclein in Lewy bodies. Nature.

[bb0280] Stewart R., Liolitsa D. (1999). Type 2 diabetes mellitus, cognitive impairment and dementia. Diabet. Med..

[bb0285] Subramaniam S.R., Ellis E.M. (2013). Neuroprotective effects of umbelliferone and esculetin in a mouse model of Parkinson's disease. J. Neurosci. Res..

[bb0290] Subramaniam S.R., Ellis E.M. (2016). Umbelliferone and esculetin protect against N-nitrosodiethylamine-induced hepatotoxicity in rats. Cell Biol. Int..

[bb0295] Tang F.L., Liu W., Hu J.X., Erion J.R., Ye J., Mei L., Xiong W.C. (2015). VPS35 deficiency or mutation causes dopaminergic neuronal loss by impairing mitochondrial fusion and function. Cell Rep..

[bb0300] Tsuyama T., Kishikawa J., Han Y.W., Harada Y., Tsubouchi A., Noji H., Kakizuka A., Yokoyama K., Uemura T., Imamura H. (2013). In vivo fluorescent adenosine 5′-triphosphate (ATP) imaging of Drosophila melanogaster and Caenorhabditis elegans by using a genetically encoded fluorescent ATP biosensor optimized for low temperatures. Anal. Chem..

[bb0305] Valente E.M., Abou-Sleiman P.M., Caputo V., Muqit M.M., Harvey K., Gispert S., Ali Z., Turco D.D., Bentivoglio A.R., Healy D.G. (2004). Hereditary early-onset Parkinson's disease caused by mutations in PINK1. Science.

[bb0310] Vilariño-Güell C., Wider C., Ross O.A., Dachsel J.C., Kachergus J.M., Lincoln S.J., Soto-Ortolaza A.I., Cobb S.A., Wilhoite G.J., Bacon J.A. (2011). VPS35 mutations in Parkinson disease. Am. J. Hum. Genet..

[bb0315] Wang J., Lu M.L., Dai H.L., Zhang S.P., Wang H.X., Wei N. (2015). Esculetin, a coumarin derivative, exerts in vitro and in vivo antiproliferative activity against hepatocellular carcinoma by initiating a mitochondrial-dependent apoptosis pathway. Braz. J. Med. Biol. Res..

[bb0320] Wang W., Wang X., Fujioka H., Hoppel C., Whone A.L., Caldwell M.A., Cullen P.J., Liu J., Zhu X. (2016). Parkinson's disease-associated mutant VPS35 causes mitochondrial dysfunction by recycling DLP1 complexes. Nat. Med..

[bb0325] Watts G.D., Wymer J., Kovach M.J., Mehta S.G., Mumm S., Darvish D., Pestronk A., Whyte M.P., Kimonis V.E. (2004). Inclusion body myopathy associated with Paget disease of bone and frontotemporal dementia is caused by mutant valosin-containing protein. Nat. Genet..

[bb0330] Westermann B. (2010). Mitochondrial fusion and fission in cell life and death. Nat. Rev. Mol. Cell Biol..

[bb0335] Yamamoto Y., Hasegawa H., Tanaka K., Kakizuka A. (2001). Isolation of neuronal cells with high processing activity for the Machado-Joseph disease protein. Cell Death Differ..

[bb0340] Youle R.J., Narendra D.P. (2011). Mechanisms of mitophagy. Nat. Rev. Mol. Cell Biol..

[bb0345] Zimprich A., Benet-Pagès A., Struhal W., Graf E., Eck S.H., Offman M.N., Haubenberger D., Spielberger S., Schulte E.C., Lichtner P. (2011). A mutation in VPS35, encoding a subunit of the retromer complex, causes late-onset Parkinson disease. Am. J. Hum. Genet..

[bb0350] Zinszner H., Kuroda M., Wang X., Batchvarova N., Lightfoot R.T., Remotti H., Stevens J.L., Ron D. (1998). CHOP is implicated in programmed cell death in response to impaired function of the endoplasmic reticulum. Genes Dev..

