# Supplementary material for: ATP Maintenance via Two Types of ATP Regulators Mitigates Pathological Phenotypes in Mouse Models of Parkinson's Disease
Source: eBioMedicine. 2017 Jul 25;22:225–41. doi: 10.1016/j.ebiom.2017.07.024 (PMC5552266; doi:10.1016/j.ebiom.2017.07.024)
Supplement: Supplementary file 1 — Supplementary Materials. [file mmc1.pdf]

Supplementary Materials for

ATP maintenance via two types of ATP regulators mitigates pathological phenotypes in mouse models of Parkinson's disease.

Masaki Nakano, Hiromi Imamura, Norio Sasaoka, Masamichi Yamamoto, Norihito Uemura, Toshiyuki Shudo, Tomohiro Fuchigami, Ryosuke Takahashi, Akira Kakizuka*

*Corresponding author. Email: kakizuka@lif.kyoto-u.ac.jp

This PDF file includes:

Fig. S1. Quantification of OCR experiments.
Fig. S2. Cell death assay based on measuring LDH in the experimental conditions in Fig. 3.
Fig. S3. Cell death assay based on measuring LDH in the experimental conditions in Fig. 4.
Fig. S4. KUSs and esculetin prevent MPP+-induced ATP depletion, ER stress, and cell death in fully differentiated PC12 cells.
Fig. S5. Additive efficacies of KUS121 and esculetin in the MPP+-induced Parkinson's disease cell culture model.
Fig. S6. Effects of KUSs and esculetin on calcium levels in neuronally differentiated PC12 cells.
Fig. S7. Cell death assay based on LDH detection in the experimental conditions in Fig. 5e and f.
Fig. S8. Time course of the effects of KUSs and esculetin on MPP+-induced ATP depletion in neuronally differentiated PC12 cells.
Fig. S9. Efficacies of KUSs and esculetin on mitochondrial ATP in the MPP+-induced Parkinson's disease cell culture model.
Fig. S10. Effects of KUSs on estrogen receptor-related receptors (ERRs)-mediated transcription.
Fig. S11. Efficacies of KUSs and esculetin on mitochondrial mass, ROS levels, and mitochondrial membrane potential in the MPP+-induced Parkinson's disease cell culture model.
Fig. S12. Effects of KUSs and esculetin on levels of proteins implicated in mitochondrial fusion, mitochondrial fission, and autophagy (mitophagy) in the MPP+-induced Parkinson's disease cell culture model.
Fig. S13. Effects of KUS121 and esculetin on MPTP-induced Parkinson's disease model mice.
Fig. S14. Tyrosine hydroxylase protein levels in midbrains in MPTP-induced Parkinson's disease model mice.
Fig. S15. Efficacies of KUS121 and esculetin on Rotenone-induced Parkinson's disease model mice.

Fig. S16. An enlarged view of triple labeling of dopaminergic neurons.

Fig. S17. Time course of KUSs and esculetin prevention of MPP+-induced cell death in neuronally differentiated PC12 cells.

Fig. S18. Time course of KUSs and esculetin effects on AMPK phosphorylation and ER stress in neuronally differentiated PC12 cells.

Fig. S19. Rotarod test at different time points.

ADDITIONAL MATERIALS AND METHODS

ADDITIONAL REFERENCES

SUPPLEMENTARY MATERIALS

Fig. S1.

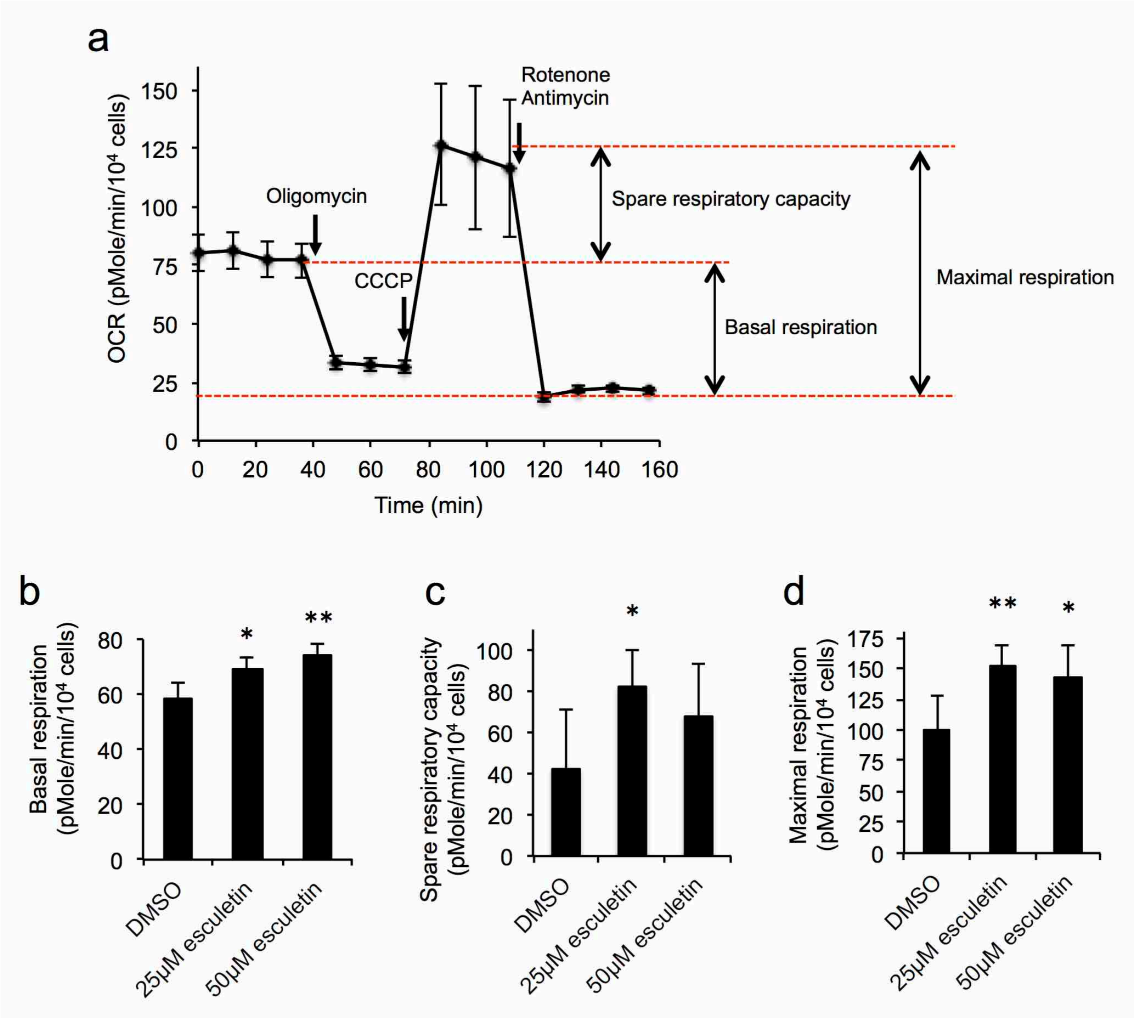

Fig. S1. Quantification of OCR experiments.

(a) Schematic drawing of the OCR values obtained from the flux analyses.

(b-d) Basal respiration (b), spare respiratory capacity (c), and maximal respiration (d) presented in Fig. 2e are shown. * $P < 0.05$, ** $P < 0.01$, ANOVA with Games-Howell post-hoc test (n = 7), vs. DMSO.

Fig. S2.

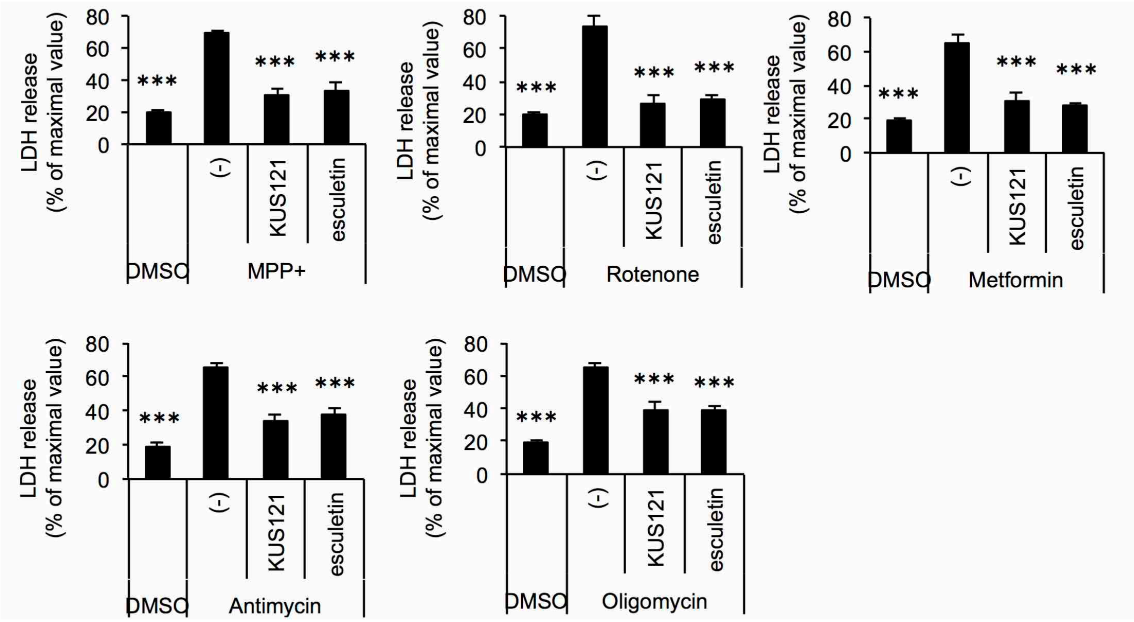

Fig. S2. Cell death assay based on measuring LDH in the experimental conditions in Fig. 3.

(a) Effects of KUS121 and esculetin on the prevention of cell death induced by mitochondrial respiratory chain complex inhibitors. Neuronally differentiated PC12 cells were incubated for 24 hours in the absence (DMSO) or the presence of 50 μM KUS121 and 50 μM esculetin, and then further incubated with each of the mitochondrial respiratory chain complex inhibitors (75 μM MPP+; 10 nM rotenone; 3 mM metformin; 100 nM antimycin; 0.01 μg/ml oligomycin) for 28 hours. Then, LDH in the media, which was released from dead cells, was measured. Relative mean values are shown, with values for control wells treated for 60 min with 0.2% Tween20 to lyse all cells set at 100%. Error bars indicate standard deviations. *** $P < 0.001$, ANOVA with Games-Howell post-hoc test (n = 4), vs. MPP+, Rotenone, Metformin, Antimycin, or Oligomycin alone (-).

Fig. S3.

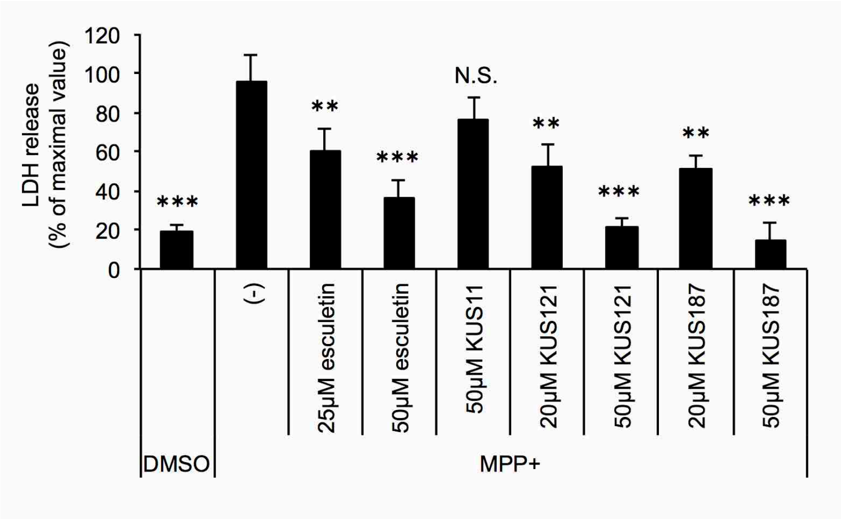

Fig. S3. Cell death assay based on measuring LDH in the experimental conditions in Fig. 4.

Effects of KUSs and esculetin on the prevention of cell death induced by MPP+. Neuronally differentiated PC12 cells were incubated for 24 hours in the absence (DMSO) or the presence of KUSs (20, or 50 µM) or esculetin (25, or 50 µM), and then further incubated with 75 µM MPP+ for 28 hours. Then, LDH in the media, which was released from dead cells, was measured. Relative mean values are shown, with values for control wells treated for 60 min with 0.2% Tween20 to lyse all cells set at 100%. Error bars indicate standard deviations. N.S., not significant. ** $P < 0.01$, *** $P < 0.001$, ANOVA with Tukey's post-hoc test (n = 3), vs. MPP+ alone (-).

Fig. S4.

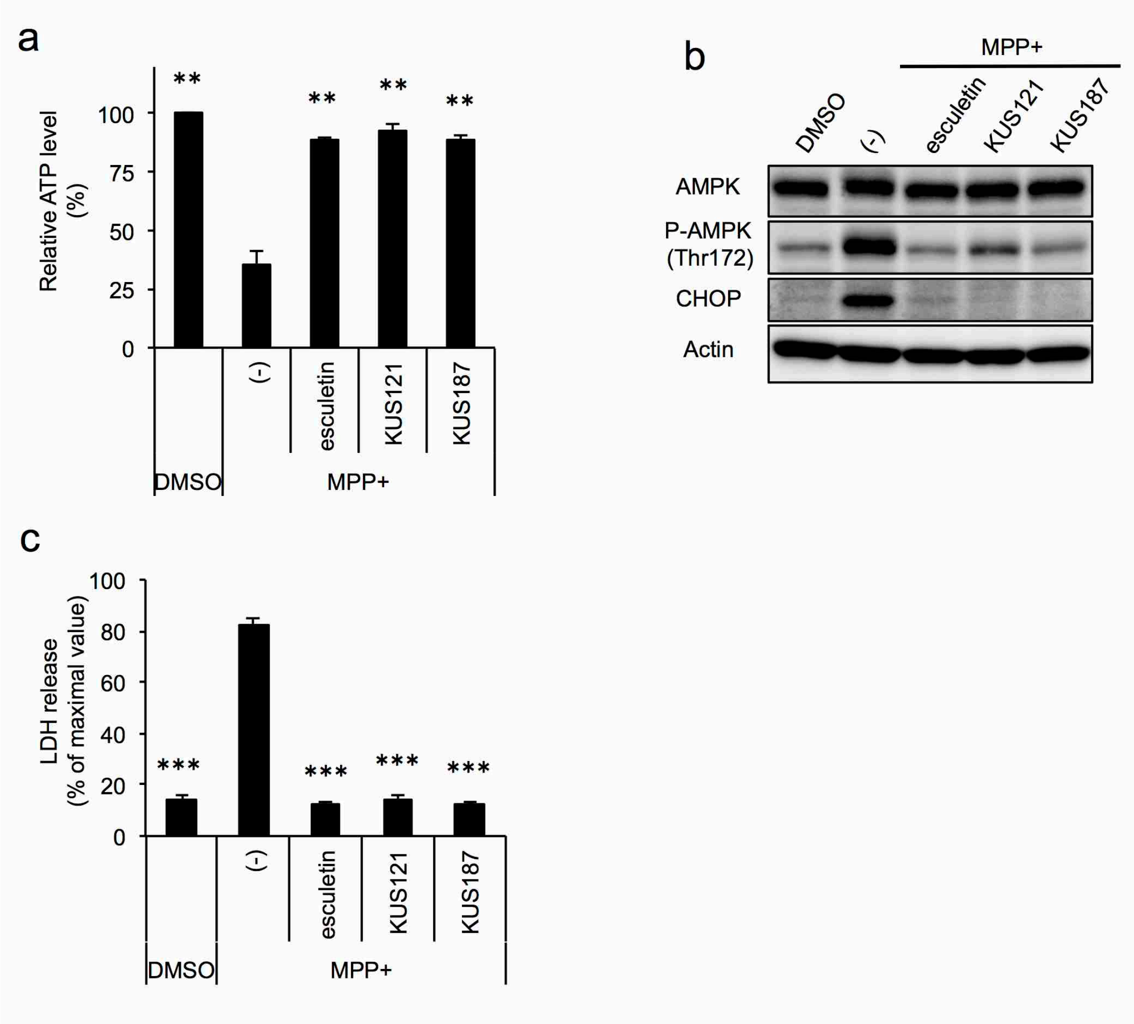

Fig. S4. KUSs and esculetin prevent MPP+-induced ATP depletion, ER stress, and cell death in fully differentiated PC12 cells.

(a) Effects of KUSs and esculetin on the prevention of ATP depletion induced by MPP+ in fully differentiated PC12 cells. PC12 cells were fully differentiated by exposure to 50 ng/ml NGF for 7 days. Fully differentiated PC12 cells were incubated for 24 hours in the absence (DMSO) or the presence of 50 μM KUSs (KUS121 and KUS187) or 50 μM esculetin, and then further incubated with 75 μM MPP+ for 24 hours. Then, total ATP amounts and live cell numbers were determined. Mean values of relative ATP levels per cell were calculated and are shown, with values for DMSO set at 100%. Error bars indicate standard deviations. ** $P < 0.01$, ANOVA with Games-Howell post-hoc test (n = 3), vs. MPP+ alone (-).

(b) Effects of KUSs and esculetin on the prevention of AMPK phosphorylation and ER stress induced by 75 μM MPP+ in fully differentiated PC12 cells. Fully differentiated PC12 cells were cultured with the conditions shown in (a), and were subjected to western blot analyses. Actin served as a loading control.

(c) Effects of KUSs and esculetin on the prevention of cell death induced by MPP+ in fully differentiated PC12 cells. In the absence (DMSO) or the presence of 50 μM KUSs or 50 μM esculetin, fully differentiated PC12 cells were incubated for 24 hours, and then further incubated with 75 μM MPP+ for 28 hours. Then, LDH in the media, which was released from dead cells, was measured. Relative mean values are shown, with values for control wells treated for 60 min with 0.2% Tween20 to lyse all cells set at 100%. Error bars indicate standard deviations. *** $P < 0.001$, ANOVA with Games-Howell post-hoc test (n = 4), vs. MPP+ alone (-).

Fig. S5.

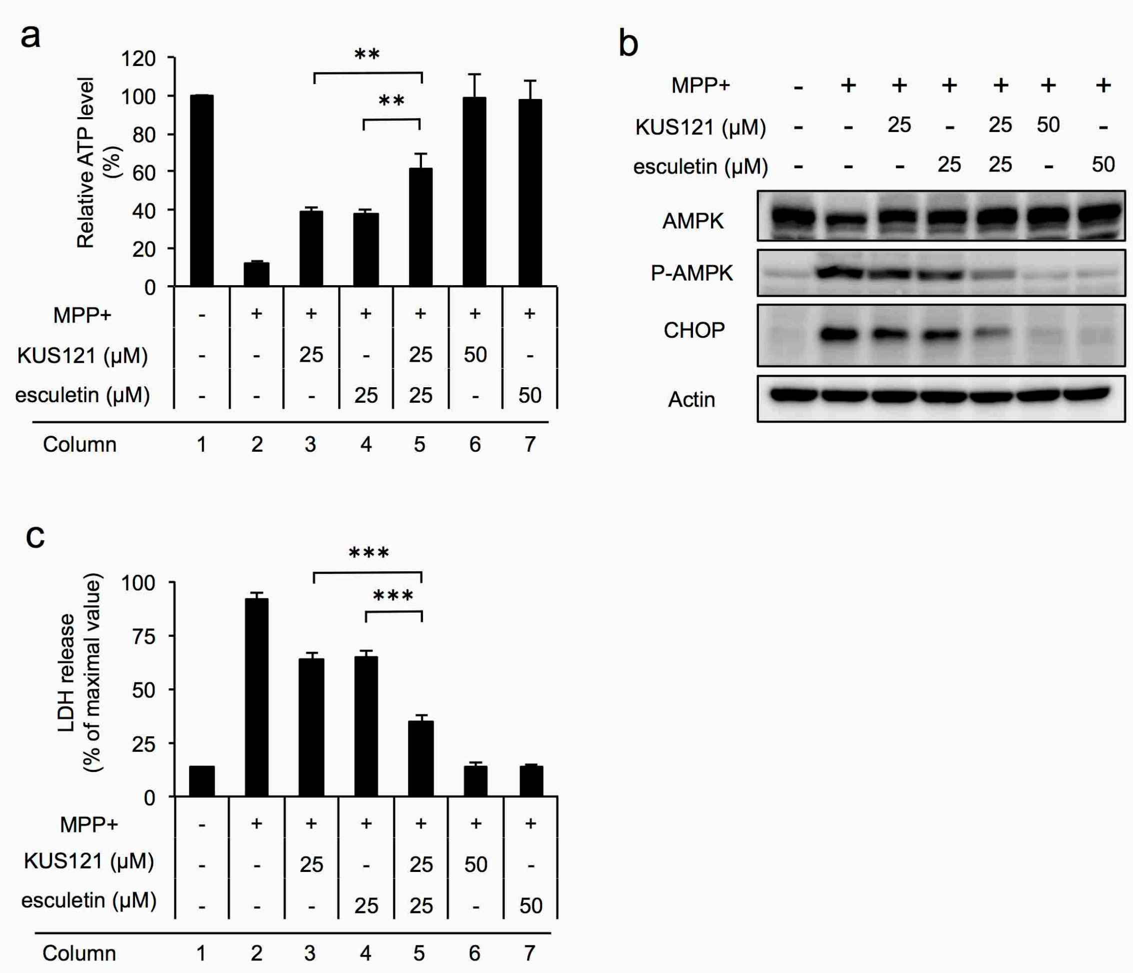

Fig. S5. Additive efficacies of KUS121 and esculetin in the MPP+-induced Parkinson's disease cell culture model.

(a) Additive effects of KUS121 and esculetin on the prevention of ATP decreases induced by MPP+. Neuronally differentiated PC12 cells were incubated for 24 hours in the absence (-) or the presence of KUS121 alone (25 μM or 50 μM), esculetin alone (25 μM or 50 μM), or a combination of KUS121 (25 μM) and esculetin (25 μM), and then further incubated with 75 μM MPP+ for 24 hours. Then, total ATP amounts and live cell numbers were determined. Mean values of relative ATP levels per cell were calculated and are shown, with values for column 1 set at 100%. Error bars indicate standard deviations. Statistical significances are shown between column 5 and column 3 or 4. ** $P < 0.01$, ANOVA with Games-Howell post-hoc test (n = 3).

(b) Additive effects of KUS121 and esculetin on the prevention of AMPK phosphorylation and ER stress induced by MPP+. Neuronally differentiated PC12 cells were cultured with the conditions shown in (a), and were subjected to western blot analyses. Actin served as a loading control.

(c) Additive effects of KUS121 and esculetin on the prevention of cell death induced by MPP+. Neuronally differentiated PC12 cells were incubated for 24 hours in the absence (-) or the presence of KUS121 alone (25 μM or 50 μM), esculetin alone (25 μM or 50 μM), or a combination of KUS121 (25 μM) and esculetin (25 μM), and then further incubated with 75 μM MPP+ for 28 hours. Then, LDH in the media, which was released from dead cells, was measured. Relative mean values are shown, with values for control wells treated for 60 min with 0.2% Tween20 to lyse all cells set at 100%. Error bars indicate standard deviations. Statistical significances are shown between column 5 and column 3 or 4. *** $P < 0.001$, ANOVA with Games-Howell post-hoc test (n = 4).

Fig. S6.

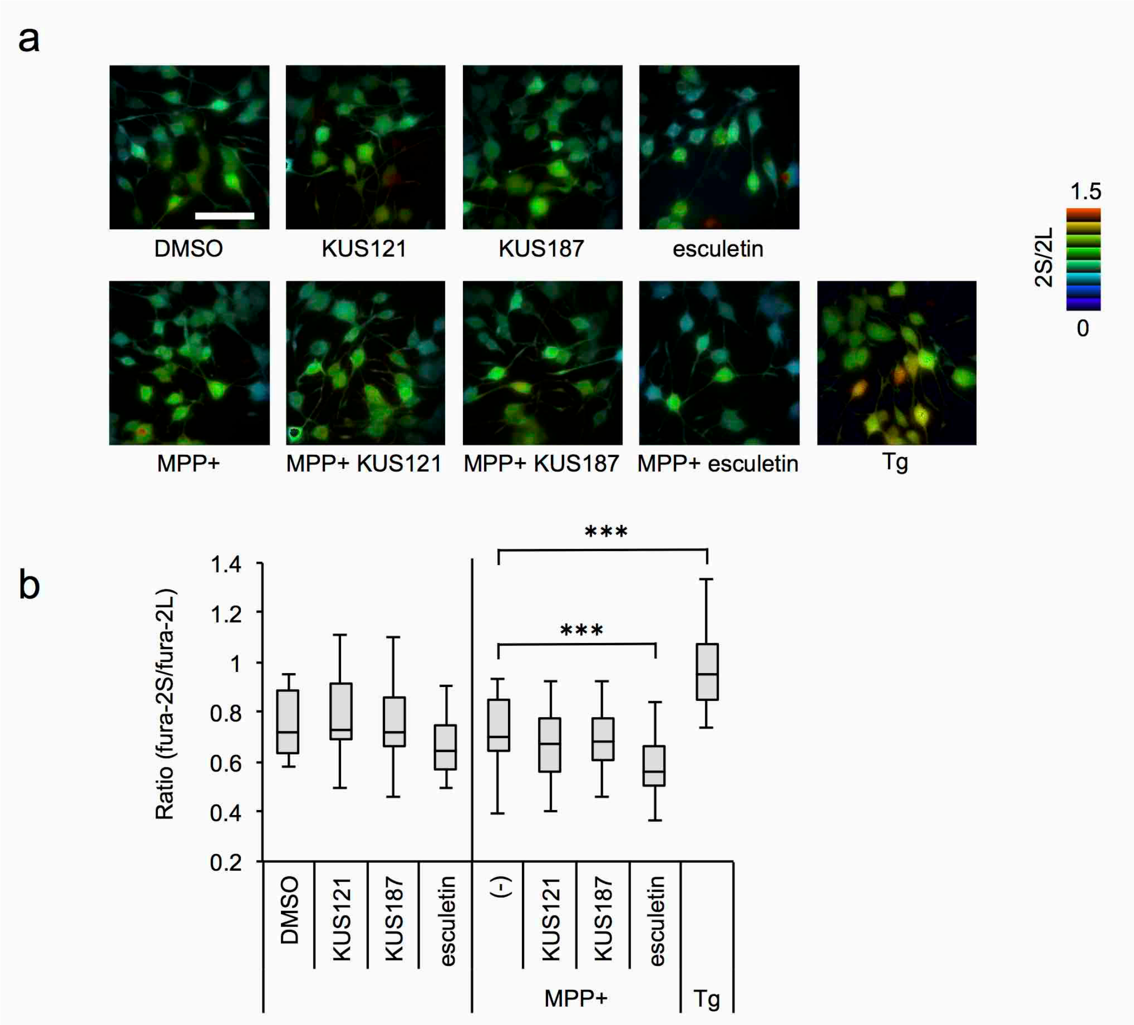

Fig. S6. Effects of KUSs and esculetin on calcium levels in neuronally differentiated PC12 cells.

(a) Representative ratio images of fura-2-AM (fura-2S (340ex)/fura-2L (387ex) emission ratios). Neuronally differentiated PC12 cells were incubated for 24 hours in the absence (DMSO) or the presence of 50 μM KUSs (KUS121 and KUS187) or 50 μM esculetin, and then further incubated with 75 μM MPP+ for 24 hours. As a positive control, cells were treated with 1 μM thapsigargin (Tg). For measurement of intracellular calcium concentrations, fura-2-AM (Dojindo, Tokyo, Japan) solution containing a PowerLoading concentrate (Invitrogen) diluted in a Recording medium (20 mM HEPES, 115 mM NaCl, 5.4 mM KCl, 0.8 mM $MgCl_2$-$6H_2O$, 1.8 mM $CaCl_2$, 5.6

mM D-glucose, pH 7.4) was added to a final concentration of 1 μM for 60 min, and cells were incubated in a fresh Recording medium. Cells were subjected to fluorescence microscopy analyses. The fura-2S/fura-2L emission ratio was calculated by dividing fura-2S intensity by fura-2L intensity for each cell. Scale bar, 200 μm.

(b) Distributions of fura-2S/fura-2L ratios of the fura-2-AM dye. The ratios were calculated from the fluorescent images in (a). Images of cells were analyzed from each condition, as follows: DMSO, 37 cells; KUS121, 25 cells; KUS187, 43 cells; esculetin, 53 cells; MPP+, 42 cells; MPP+ KUS121, 50 cells; MPP+ KUS187, 44 cells; MPP+ esculetin, 34 cells; Tg as a positive control, 33 cells. Distributions of fura-2S/fura-2L ratios are shown as box-and-whisker plots. The black bars indicate the median, boxes are 25th-75th quartiles, and whiskers indicate the minimum and maximum values. Statistical significances are shown among MPP+-treated and Tg-treated groups. *** $P < 0.001$, ANOVA with Tukey's post-hoc test, vs. MPP+ alone (-).

Fig. S7.

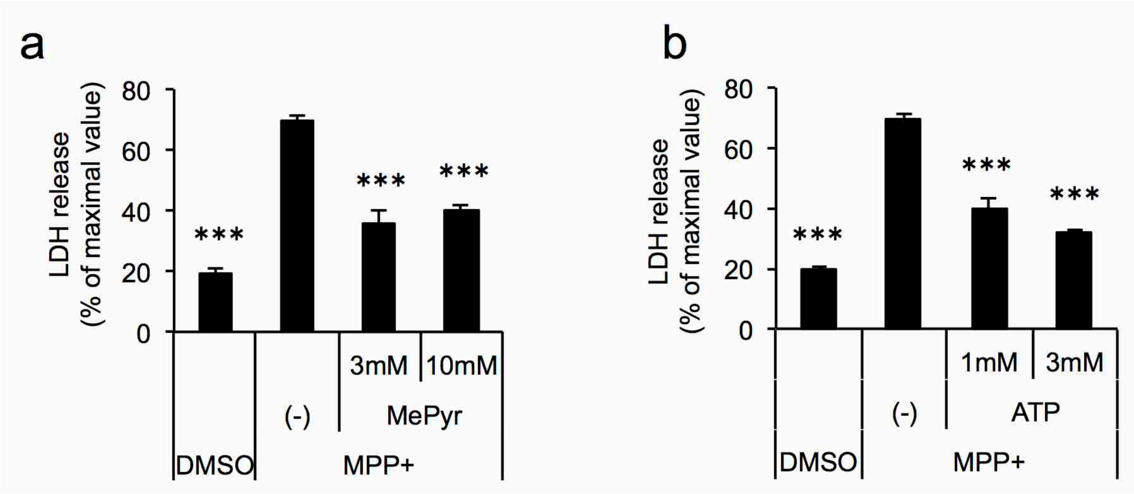

Fig. S7. Cell death assay based on LDH detection in the experimental conditions in Fig. 5e and f.

(a, b) Effects methylpyruvate (MePyr) or ATP treatment on the prevention of cell death induced by MPP+. Neuronally differentiated PC12 cells were cultured with the conditions shown in (Fig. 5e, f). Then, LDH in the media, which was released from dead cells, was measured. Relative mean values are shown, with values for control wells treated for 60 min with 0.2% Tween20 to lyse all cells set at 100%. Error bars indicate standard deviations. *** $P < 0.001$, ANOVA with Games-Howell post-hoc test (n = 4), vs. MPP+ alone (-).

Fig. S8.

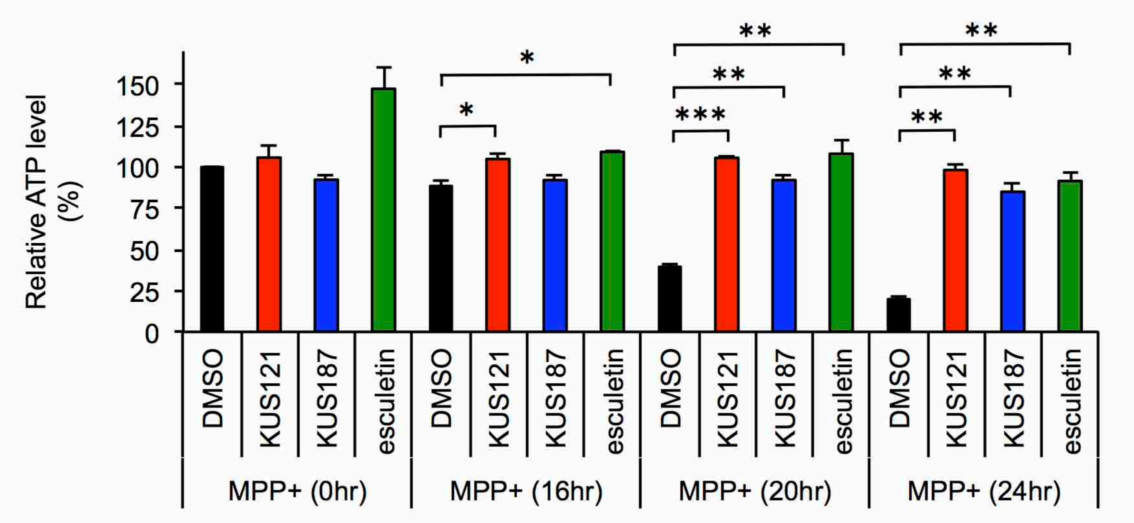

Fig. S8. Time course of the effects of KUSs and esculetin on MPP+-induced ATP depletion in neuronally differentiated PC12 cells.

(a) Effects of KUSs and esculetin in the prevention of ATP depletion induced by MPP+ over time (0, 16, 20, 24 hours). Neuronally differentiated PC12 cells were incubated for 24 hours in the absence (DMSO) or the presence of 50 μM KUSs (KUS121 and KUS187) or 50 μM esculetin, and then further incubated with 75 μM MPP+ for 0, 16, 20, and 24 hours. Then, total ATP amounts and live cell numbers were determined. Mean values of relative ATP levels per cell were calculated and are shown, with values for MPP+ (DMSO) at 0 hour set at 100%. Error bars indicate standard deviations. * $P < 0.05$, ** $P < 0.01$, *** $P < 0.001$, ANOVA with Games-Howell post-hoc test (n = 3), vs. MPP+ alone (DMSO).

Fig. S9.

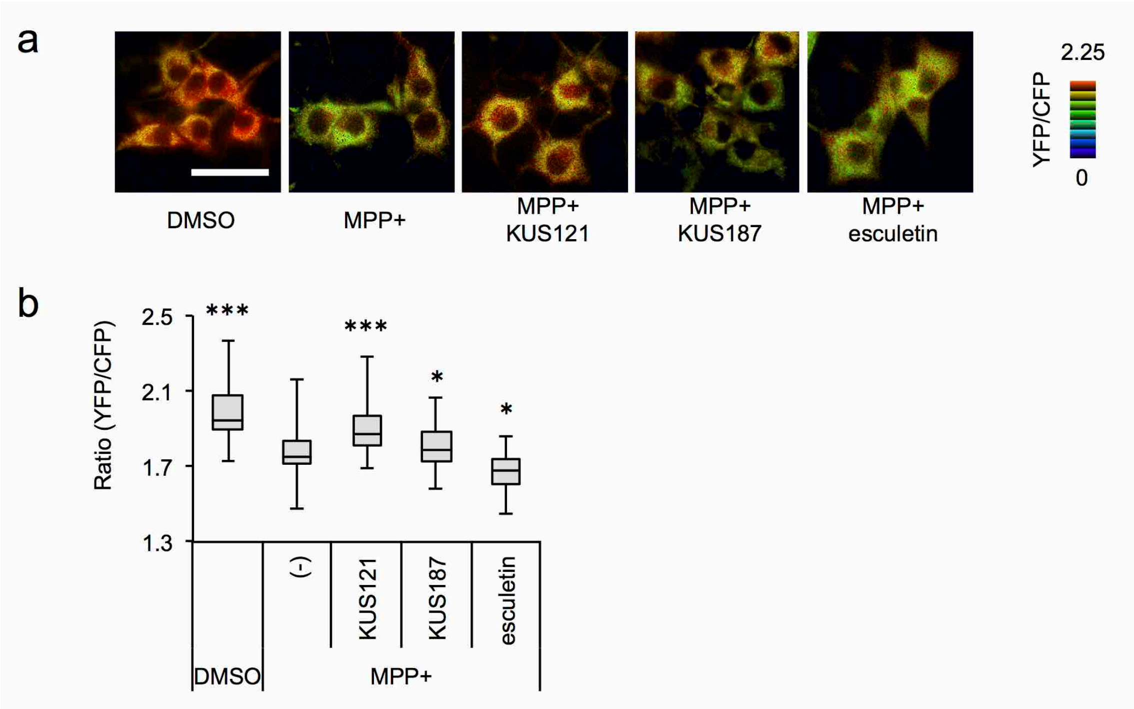

Fig. S9. Efficacies of KUSs and esculetin on mitochondrial ATP in the MPP+-induced Parkinson's disease cell culture model.

(a) Representative ratiometric pseudocolor FRET images of mit-ATeam1.03-expressing PC12 cells. Neuronally differentiated PC12 cells expressing mit-ATeam1.03 were incubated for 24 hours in the absence (DMSO) or the presence of 50 µM KUSs (KUS121 and KUS187) or 50 µM esculetin, and then further incubated with 75 µM MPP+ for 24 hours. YFP/CFP emission ratios of mit-ATeam1.03 were then calculated for individual cells in captured images. Scale bar, 50 µm.

(b) Distributions of YFP/CFP emission ratios of mit-ATeam1.03. The ratios were calculated from the fluorescent images in (a). Distributions of YFP/CFP emission ratios are shown as box-and-whisker plots. The black bars indicate the median, boxes are 25th-75th quartiles, and whiskers indicate the minimum and maximum values. DMSO, 44 cells; MPP+, 41 cells; MPP+ KUS121, 35 cells; MPP+ KUS187, 47 cells; MPP+ esculetin, 34 cells. * $P < 0.05$, *** $P < 0.001$, ANOVA with Tukey's post-hoc test, vs. MPP+ alone (-).

Fig. S10.

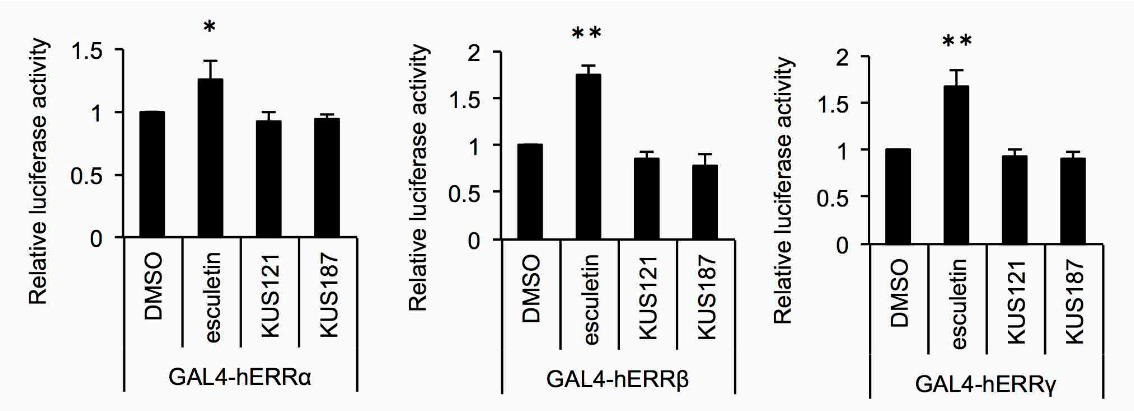

Fig. S10. Effects of KUSs on estrogen receptor-related receptors (ERRs)-mediated transcription.

Transcriptional activation profiles of ERRα, β, γ in response to 50 μM of KUS121, KUS187, or esculetin were performed as diagrammed in Fig. 1a. Mean values of relative luciferase activities, after normalization with β-galactosidase activities, are shown, with values for DMSO alone set at 1.0. Error bars indicate standard deviations. * $P < 0.05$, ** $P < 0.01$, ANOVA with Games-Howell post-hoc test (n = 4), vs. DMSO.

Fig. S11.

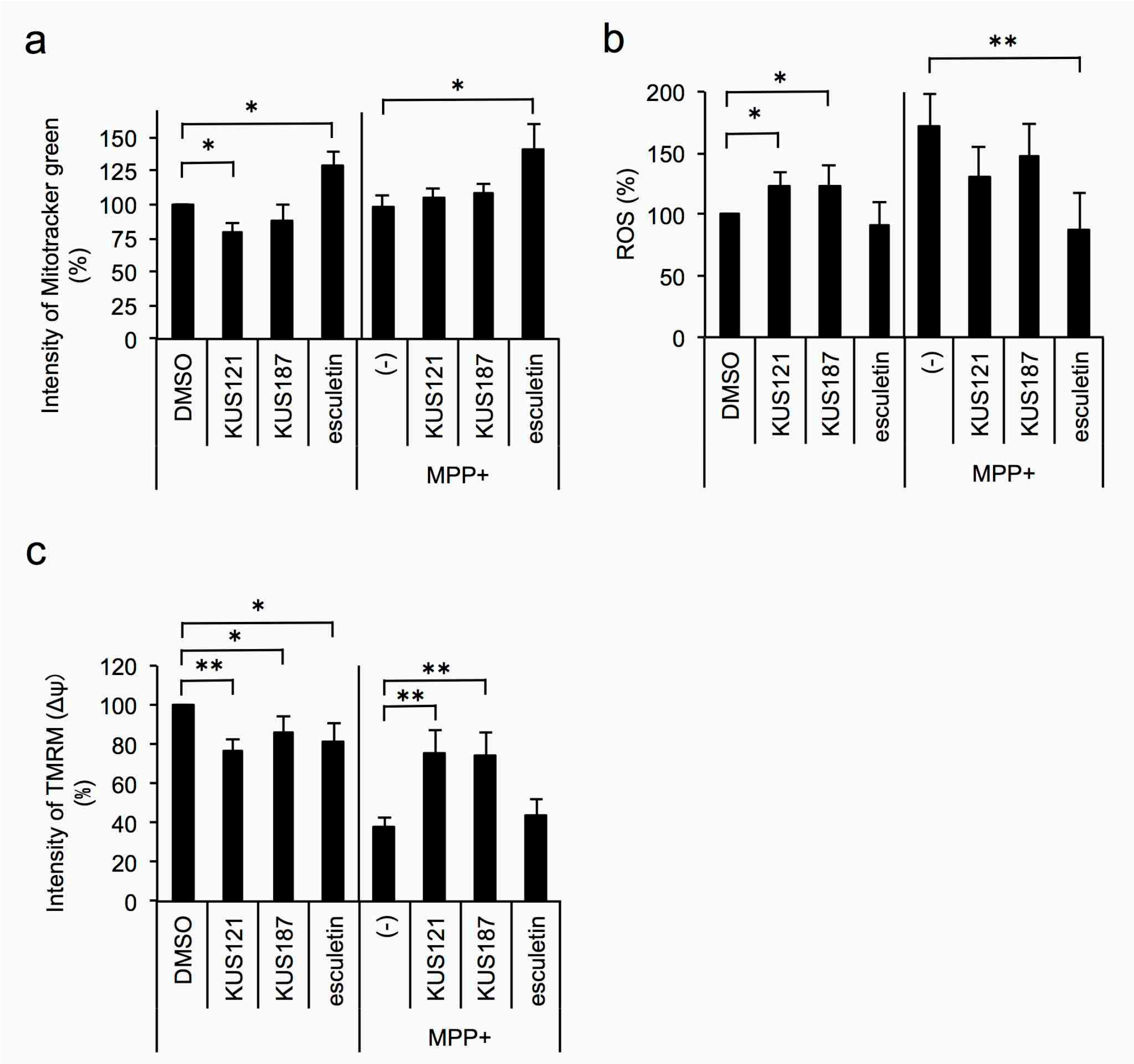

Fig. S11. Efficacies of KUSs and esculetin on mitochondrial mass, ROS levels, and mitochondrial membrane potential in the MPP+-induced Parkinson's disease cell culture model.

(a) Detection of MitoTracker Green FM fluorescence levels with a FACS Calibur flow cytometer. Neuronally differentiated PC12 cells were incubated for 24 hours in the absence (DMSO) or the presence of 50 μM KUS121, 50 μM KUS187, or 50 μM esculetin, and then further incubated with 75 μM MPP+ for 24 hours. Cells were incubated with 50 nM MitoTracker Green FM, and fluorescence was monitored with a FACS Calibur flow cytometer. Mean values of relative fluorescence intensities are shown as mitochondrial masses, with values for DMSO alone set at 100%. Error bars

indicate standard deviations. * $P < 0.05$, ANOVA with Games-Howell post-hoc test (n = 5), vs. DMSO (non-MPP+-treated group). * $P < 0.05$, ANOVA with Games-Howell post-hoc test (n = 5), vs. MPP+ alone (-) (MPP+-treated group).

(b) Detection of ROS with a FACS Calibur flow cytometer. Neuronally differentiated PC12 cells were cultured with the conditions shown in (a). Afterward, cells were incubated in serum-free medium containing 10 μM H_2DCFDA, and fluorescence was monitored with a FACS Calibur flow cytometer. Mean values of relative fluorescence intensities are shown, with values for DMSO alone set at 100%. Error bars indicate standard deviations. * $P < 0.05$, ANOVA with Games-Howell post-hoc test (n = 6), vs. DMSO (non-MPP+-treated group). ** $P < 0.01$, ANOVA with Games-Howell post-hoc test (n = 6), vs. MPP+ alone (-) (MPP+-treated group).

(c) Detection of TMRM fluorescence levels with a FACS Calibur flow cytometer. Neuronally differentiated PC12 cells were cultured with the conditions shown in (a), and were incubated with 50 nM TMRM; fluorescence was monitored with a FACS Calibur flow cytometer. Mean values of relative fluorescence intensities are shown as the mitochondrial membrane potential ($\Delta\psi$), with values for DMSO alone set at 100%. Error bars indicate standard deviations. * $P < 0.05$, ** $P < 0.01$, ANOVA with Games-Howell post-hoc test (n = 5), vs. DMSO (non-MPP+-treated group). ** $P < 0.01$, ANOVA with Games-Howell post-hoc test (n = 5), vs. MPP+ alone (-) (MPP+-treated group).

Fig. S12.

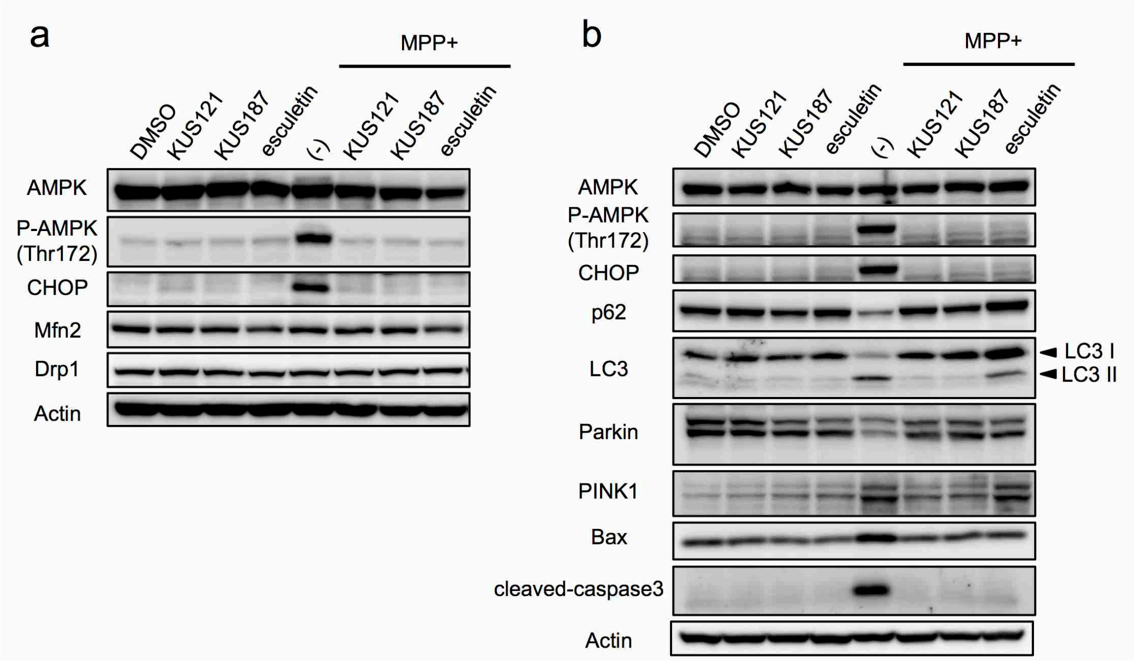

Fig. S12. Effects of KUSs and esculetin on levels of proteins implicated in mitochondrial fusion, mitochondrial fission, and autophagy (mitophagy) in the MPP+-induced Parkinson's disease cell culture model.

(a) Effects of KUSs and esculetin on levels of proteins involved in mitochondrial fusion and mitochondrial fission. Neuronally differentiated PC12 cells were incubated for 24 hours in the absence (DMSO) or the presence of 50 μM KUSs (KUS121 and KUS187) or 50 μM esculetin, and then further incubated with 75 μM MPP+ for 24 hours, and were then subjected to western blot analyses. Actin served as a loading control.

(b) Effects of KUSs and esculetin on levels of proteins involved in autophagy or mitophagy. Neuronally differentiated PC12 cells were cultured with the conditions shown in (a), and were then subjected to western blot analyses. Actin served as a loading control.

Fig. S13.

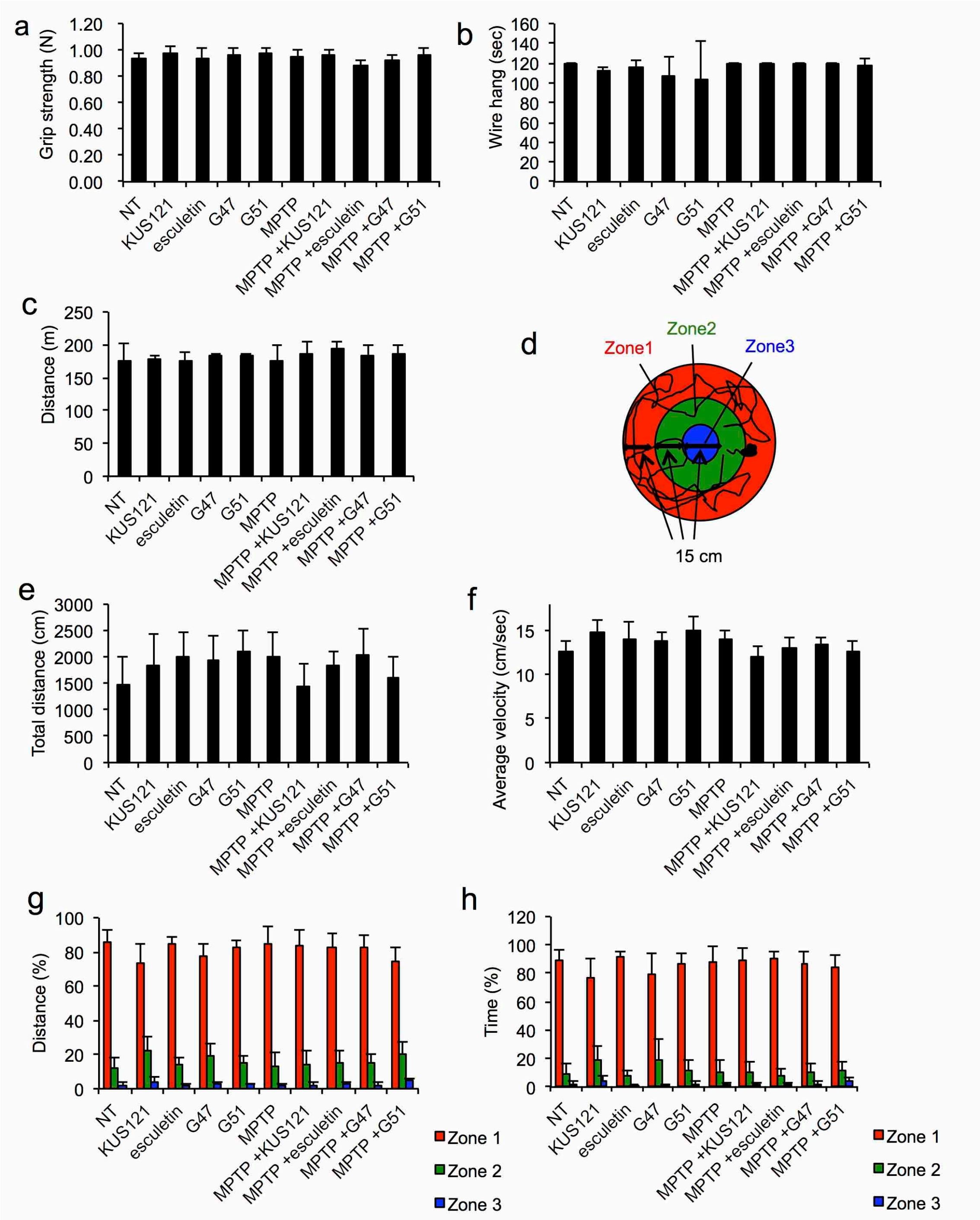
a
Grip strength (N)
1.20
1.00
0.80
0.60
0.40
0.20
0.00
NT
KUS121
esculetin
G47
G51
MPTP
MPTP +KUS121
MPTP +esculetin
MPTP +G47
MPTP +G51
b
Wire hang (sec)
160
140
120
100
80
60
40
20
0
c
Distance (m)
250
200
150
100
50
0
d
Zone1
Zone2
Zone3
15 cm
e
Total distance (cm)
3000
2500
2000
1500
1000
500
0
f
Average velocity (cm/sec)
15
10
5
0
g
Distance (%)
100
80
60
40
20
0
Zone 1
Zone 2
Zone 3
h
Time (%)
120
100
80
60
40
20
0
Zone 1
Zone 2
Zone 3

Fig. S13. Effects of KUS121 and esculetin on MPTP-induced Parkinson's disease model mice.

(a) The grip strength test was performed, and mean values of forelimb grip strength (newton (N)) with standard deviations are shown. No significant difference was observed among the groups.

(b) The wire hang test was performed, and mean times (sec) to fall with standard deviations are shown. No significant difference was observed among the groups.

(c) The treadmill test was performed, and no significant difference was observed among the groups. Mice were allowed to rest in the treadmill for 5 min and were then exercised by running 3 min at 10 m/min, 2 min at 15 m/min, 2 min at 20 m/min, then 4 min at 25 m/min at 0% incline. (a-c) All groups of mice are shown: no drug-treated control (NT; n = 7); KUS121-treated (KUS121; n = 6); esculetin-treated (esculetin; n = 6); GSK4716-treated (G47; n = 5); GSK5182-treated (G51; n = 5); MPTP-treated (MPTP; n = 7); MPTP + KUS121-treated (MPTP +KUS121; n = 7); MPTP + esculetin-treated (MPTP +esculetin; n = 7); MPTP + GSK4716-treated (MPTP +G47; n = 7); and MPTP + GSK5182-treated (MPTP +G51; n = 7).

(d-h) An open field test was performed, and mean values with standard deviations are shown. No significant difference was observed among the groups in all examinations. The partitions of the zones are indicated (d). The parameters measured were: total distance (e), the average velocity of movement (f), the percentage of the distance covered in each zone (g), the percentage of the time during which the mouse moved or stayed in each zone (h). (e-h) All groups of mice are shown: no drug-treated control (NT; n = 6); KUS121-treated (KUS121; n = 6); esculetin-treated (esculetin; n = 6); GSK4716-treated (G47; n = 5); GSK5182-treated (G51; n = 5); MPTP-treated (MPTP; n = 6); MPTP + KUS121-treated (MPTP +KUS121; n = 4); MPTP + esculetin-treated (MPTP +esculetin; n = 4); MPTP + GSK4716-treated (MPTP +G47; n = 4); and MPTP + GSK5182-treated (MPTP +G51; n = 4).

Fig. S14.

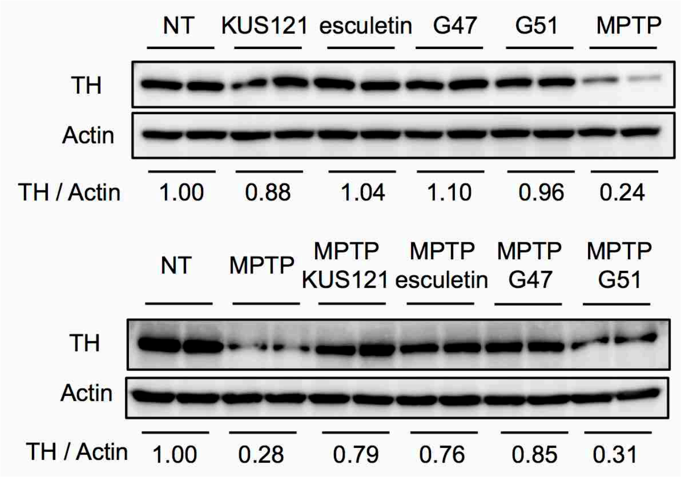

Fig. S14. Tyrosine hydroxylase protein levels in midbrains in MPTP-induced Parkinson's disease model mice.

Western blot analyses of mouse midbrains, containing the striatum and the substantia nigra, using an anti-tyrosine hydroxylase (TH) antibody. Actin served as a loading control. No drug-treated control (NT), KUS121-treated (KUS121), esculetin-treated (esculetin), GSK4716-treated (G47), GSK5182-treated (G51). MPTP-treated (MPTP), MPTP + KUS121-treated (MPTP +KUS121), MPTP + esculetin-treated (MPTP +esculetin), MPTP + GSK4716-treated (MPTP +G47), MPTP + GSK5182-treated (MPTP +G51). Mean ratios of TH/Actin from two mice for each respective treatment are shown, with values for NT set at 1.00.

Fig. S15.

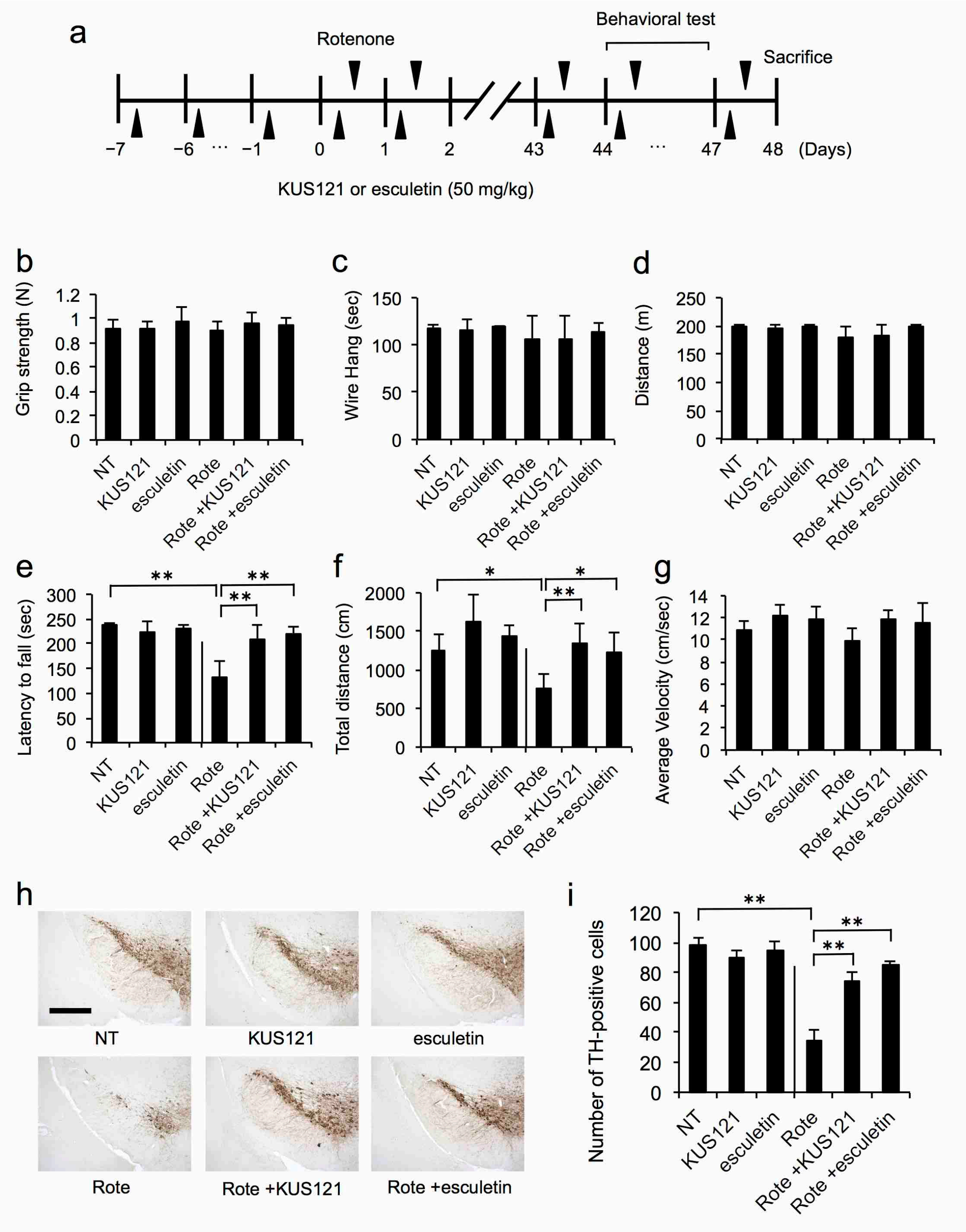
a
Behavioral test
Rotenone
Sacrifice
−7
−6
−1
0
1
2
43
44
47
48
(Days)
KUS121 or esculetin (50 mg/kg)
b
Grip strength (N)
c
Wire Hang (sec)
d
Distance (m)
e
Latency to fall (sec)
f
Total distance (cm)
g
Average Velocity (cm/sec)
NT
KUS121
esculetin
Rote
Rote +KUS121
Rote +esculetin
h
i
Number of TH-positive cells

Fig. S15. Efficacies of KUS121 and esculetin on Rotenone-induced Parkinson's disease model mice.

(a) Schematic drawing of experimental schedules for induction and treatment of rotenone-induced Parkinson's disease model mice. See details in "ADDITIONAL MATERIALS AND METHODS".

(b) The grip strength test was performed, and mean values of forelimb grip strength (newton (N)) with standard deviations are shown. No significant difference was observed among the groups.

(c) The wire hang test was performed, and mean times (sec) to fall with standard deviations are shown. No significant difference was observed among the groups.

(d) The treadmill test was performed, as described for Fig. S13c. No significant difference was observed among the groups.

(e) The rotarod test was performed, and mean times (sec) to fall with standard deviations are shown. Statistical significances are shown among rotenone-treated and no drug-treated control (NT) groups. ** $P < 0.01$, ANOVA with Games-Howell post-hoc test, vs. rotenone alone (Rote).

(f, g) An open field test was performed, and mean values with standard deviations are shown. The total distance (f), and the average velocity of movement (g) were examined. Statistical significances were observed among rotenone-treated groups and no drug-treated control (NT) groups in (f). * $P < 0.05$, ** $P < 0.01$, ANOVA with Games-Howell post-hoc test, vs. rotenone alone (Rote).

(b-g) All groups of mice are shown: no drug-treated control (NT; n = 6); KUS121-treated (KUS121; n = 5); esculetin-treated (esculetin; n = 5); rotenone-treated (Rote; n = 6); rotenone + KUS121-treated (Rote +KUS121; n = 6); rotenone + esculetin-treated (Rote +esculetin; n = 6). Error bars indicate standard deviations.

(h) Immunohistochemical analyses of coronal sections of the mouse substantia nigra, stained with an anti-tyrosine hydroxylase (TH) antibody, followed by the ABC method. Representative images are shown. Scale bar, 400 μm.

(i) Quantification of TH-positive neurons in (h), using a BZ-X700 fluorescence microscope (KEYENCE). Error bars indicate standard deviations. Statistical significances are shown among rotenone-treated and no drug-treated control (NT) groups. ** $P < 0.01$, ANOVA with Games-Howell post-hoc test, (n = 3) vs. rotenone alone (Rote).

Fig. S16.

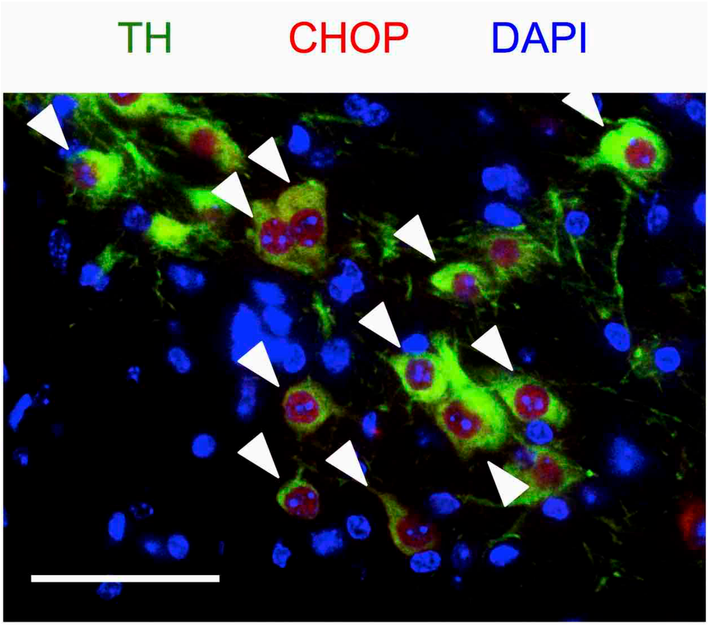

Fig. S16. An enlarged view of triple labeling of dopaminergic neurons.

An enlarged view of a triple labeling image of Fig. 10c is shown. Dopaminergic neurons of the substantia nigra from the MPTP-treated mouse were triple labeled with an anti-tyrosine hydroxylase (TH) antibody (green), an anti-CHOP antibody (red), and DAPI (blue). Scale bar, 50 μm. CHOP expression increased in the nucleus of the dopaminergic neurons in the substantia nigra pars compacta of MPTP-treated mice (white arrowheads).

Fig. S17.

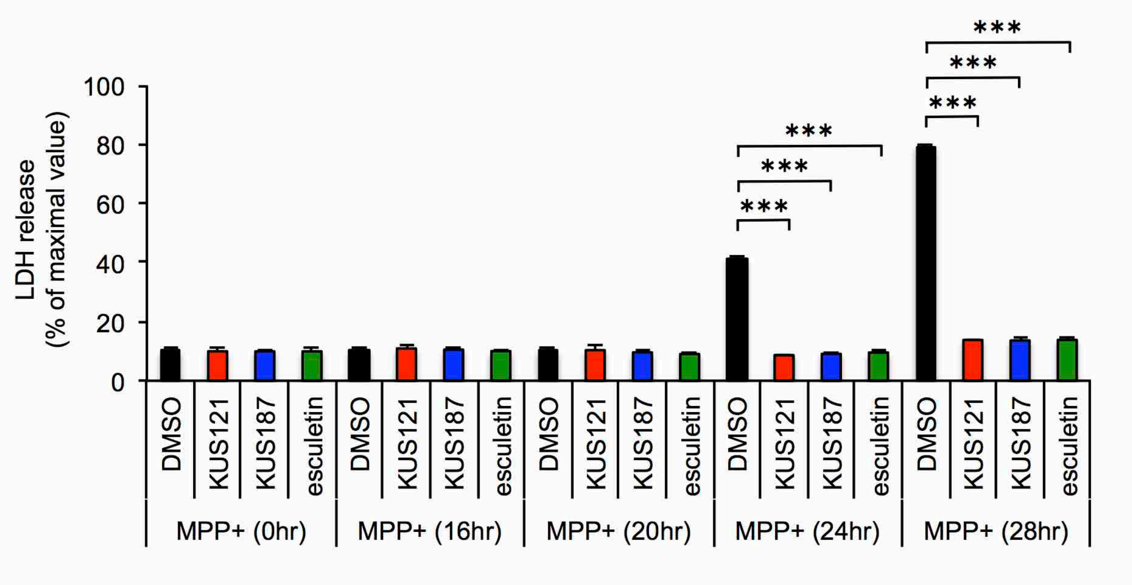

Fig. S17. Time course of KUSs and esculetin prevention of MPP+-induced cell death in neuronally differentiated PC12 cells.

Effects of KUSs (KUS121 and KUS187) and esculetin on the prevention of cell death induced by MPP+ over time. Neuronally differentiated PC12 cells were cultured as in Fig. S8, but were treated with MPP+ for 0, 16, 20, 24, and 28 hours. LDH in the media, which was released from dead cells, was then measured. Relative mean values are shown, with values for control wells treated for 60 min with 0.2% Tween20 to lyse all cells set at 100%. Error bars indicate standard deviations. *** $P < 0.001$, ANOVA with Games-Howell post-hoc test (n = 3), vs. MPP+ alone (DMSO).

Fig. S18.

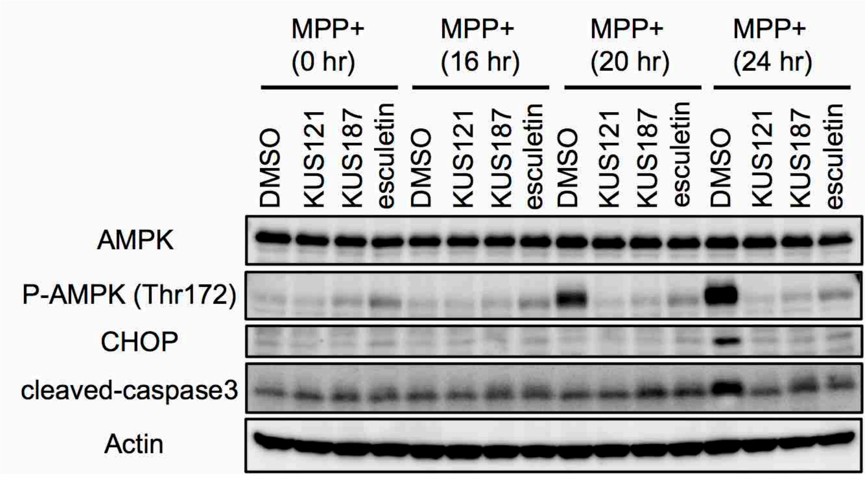

Fig. S18. Time course of KUSs and esculetin effects on AMPK phosphorylation and ER stress in neuronally differentiated PC12 cells.

Effects of KUSs (KUS121 and KUS187) and esculetin on the prevention of MPP+-induced AMPK phosphorylation and ER stress over time (0, 16, 20, 24 hours). Neuronally differentiated PC12 cells were cultured as in Fig. S8, and were then subjected to western blot analyses. Actin served as a loading control.

Fig. S19.

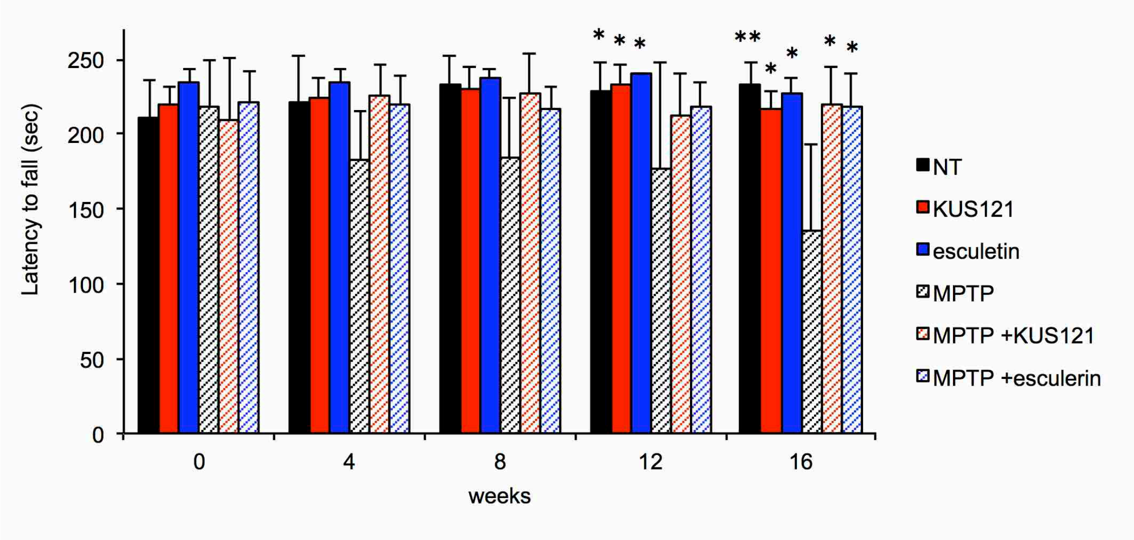

Fig. S19. Rotarod test at different time points.

The rotarod test was performed, and mean times (sec) to fall with standard deviations are shown from 0 to 20 weeks (0, 4, 8, 12, 16 weeks) after starting the experiments (refer to Fig. 8a for the treatment schedule). No drug-treated control (NT; n = 7), KUS121-treated (KUS121; n = 6), esculetin-treated (esculetin; n = 6), MPTP-treated (MPTP; n = 7), MPTP + KUS121-treated (MPTP +KUS121; n = 7), MPTP + esculetin-treated (MPTP +esculetin; n = 7). Error bars indicate standard deviations. * $P < 0.05$, ** $P < 0.01$, ANOVA with Games-Howell post-hoc test, vs. MPTP-treated group (MPTP).

ADDITIONAL MATERIALS AND METHODS

Measurement of lactate dehydrogenase (LDH) release

Release of LDH into the culture media from cells with damaged membranes was measured using a LDH-Cytotoxic Test kit (Wako, Tokyo, Japan), following the manufacturer's protocol. Briefly, 50 μL of supernatant from each well was collected to assay LDH release. The activity of LDH (the absorbance at 570 nm) was measured with an ARVO multi-label counter (Perkin Elmer, Inc., Waltham, MA, USA). The results were normalized to the maximal LDH release, which was determined by treating control wells for 60 min with 0.2% Tween20 to lyse all cells.

FACS analyses

For determination of mitochondrial membrane potential and mitochondrial mass, cells were treated with 50 nM TMRM (Invitrogen) and 50 nM MitoTracker Green FM (Invitrogen), respectively, at 37°C for 30 min before fluorescence measurements, and analyzed with a FACS Calibur flow cytometer (BD Biosciences) (Cottet-Rousselle et al., 2011). Excitation was at 488 nm, and green and red emission filters were at 530±15 nm and 585±21 nm. For intracellular ROS accumulation, cells were incubated at 37°C for 60 min with serum-free medium containing 10 μM CM-H_2DCFDA (Molecular Probes) and fluorescence was analyzed with a FACS Calibur flow cytometer with excitation at 488 nm and emission at 530±15 nm. A total of 20,000 cells were counted per sample, and the data were processed using standard software.

Grip strength test

Grip strength was tested using a grip strength meter. Mice were positioned to grab a metal mesh with only their forelimbs and were then pulled back applying uniform force until they released the mesh. The grip strength was measured three times and the averages and standard deviations of three measurements were calculated.

Wire hang test

The wire hang test was used to evaluate limb strength. The test was performed by placing the mouse on the wire bars of a standard mouse cage lid, followed by swiftly inverting the lid. Wire netting territory was a 10 cm by 10 cm square with a wire spacing of 1.5 cm. Latency to fall into the empty cage (dimensions, length 22.5 cm, width 22 cm, height 30 cm) was measured with a stopwatch over a 2-minute maximum test session. A cleaned wire lid was used for each mouse. The grip strength was measured three times and the averages and standard deviations of three measurements were calculated.

Treadmill intervention

A treadmill test was used to evaluate physical capacity and endurance. The treadmill consisted of eight parallel runways without inclination. The treadmill test began at slow speed (10 m/min), and the speed of the treadmill was gradually increased stepwise until it reached 25 m/min. These speeds were maintained until the end of the experiment. Mice were allowed to rest in the treadmill for 5 min and were then exercised by running 3 min at 10 m/min, 2 min at 15 m/min, 2 min at 20 m/min, and 4 min at 25 m/min at 0% incline.

Open-field test

Before starting the open-field test, mice were acclimated to the testing room for 60 minutes. A DCR-TRV20 (SONY, Tokyo, Japan) camera was placed above the field, and recorded the movements of the mice. The open field test was performed as described previously (Sasaoka et al., 2014). Each mouse was placed in the center of a circular box (75 cm in diameter) and was allowed to freely explore for 5 min, under a standard fluorescent light. Three zones were set in the box (zone 3, center of the circle, with a 15 cm diameter; zone 1, the outer-most annulus, with a width of 15 cm; zone 2, the annulus between zone 1 and zone 3, with a width of 15 cm). The time spent in each zone was calculated using SMART software (version 2.0, Panlab) during the test.

Rotenone-induced Parkinson's disease model mice

In the rotenone-induced Parkinson's mouse model, oral administration of KUS121 (50 mg/kg/day), esculetin (50 mg/kg/day) was performed daily, starting 1 week before rotenone injection. 8 week-old male C57BL/6N Slc mice weighing 20~25 g were used for the rotenone-induced Parkinson's disease model. Animals were housed at a controlled room temperature (25±1 °C) and humidity (54±1%) under a 12-hour light/12-hour dark cycle. Animals were allowed access to food and water ad libitum. Animals were randomly divided into six groups: No drug-treated control (NT) group (n = 6); KUS121 group (n = 5); esculetin group (n = 5); Rotenone group (n = 6); Rotenone +KUS121 group (n = 6); Rotenone +esculetin group (n = 6). The mice were orally injected with 30 mg/kg rotenone (Sigma) for 48 days. Rotenone was dissolved in 0.5% carboxymethyl cellulose sodium salt (CMC) (Sigma) to make a 10 mg/mL solution. Subsequently, rotenone solution was administered orally once per day. 0.5% CMC was administered orally to control mice as a vehicle (Inden et al., 2011).
